# A new approach to the determination of tubular membrane capacitance: passive membrane electrical properties under reduced electrical conductivity of the extracellular solution

**DOI:** 10.1007/s00424-022-02756-x

**Published:** 2022-10-14

**Authors:** Jiří Šimurda, Milena Šimurdová, Olga Švecová, Markéta Bébarová

**Affiliations:** 1 Department of Physiology, Faculty of Medicine, Masaryk University, Kamenice 5, 625 00 Brno, Czech Republic; 2 Department of Internal Medicine and Cardiology, University Hospital Brno and Faculty of Medicine, Masaryk University, Jihlavská 20, 625 00 Brno, Czech Republic

**Keywords:** Cardiomyocyte, Tubular system, Tubular membrane capacitance, Novel method, Sucrose

## Abstract

The transverse-axial tubular system (tubular system) of cardiomyocytes plays a key role in excitation–contraction coupling. To determine the area of the tubular membrane in relation to the area of the surface membrane, indirect measurements through the determination of membrane capacitances are currently used in addition to microscopic methods. Unlike existing electrophysiological methods based on an irreversible procedure (osmotic shock), the proposed new approach uses a reversible short-term intermittent increase in the electrical resistance of the extracellular medium. The resulting increase in the lumen resistance of the tubular system makes it possible to determine separate capacitances of the tubular and surface membranes. Based on the analysis of the time course of the capacitive current, computational relations were derived to quantify the elements of the electrical equivalent circuit of the measured cardiomyocyte including both capacitances. The exposition to isotonic low-conductivity sucrose solution is reversible which is the main advantage of the proposed approach allowing repetitive measurements on the same cell under control and sucrose solutions. Experiments on rat ventricular cardiomyocytes (*n* = 20) resulted in the surface and tubular capacitance values implying the fraction of tubular capacitance/area of 0.327 ± 0.018. We conclude that the newly proposed method provides results comparable to the data obtained by the currently used detubulation method and, in addition, by being reversible, allows repeated evaluation of surface and tubular membrane parameters on the same cell.

**Supplementary Information:**

The online version contains supplementary material available at 10.1007/s00424-022-02756-x.

## Introduction

Measurements of the electrical parameters characterizing cardiac cellular membranes (separating the external and internal environment of cardiomyocytes) are complicated by complex membrane geometry due to the existence of the transverse-axial tubular system. Given its physiological importance (reviewed in [3, 4]), it is desirable to investigate the properties of the surface and tubular membranes separately. An important first step is to determine the area of the surface and tubular membrane.

Cell membrane capacitance *C*_m_ measured by electrophysiological methods can be considered a measure of the cell membrane area using the relationship

1 $$C_{{\text{m}}} = \frac{\varepsilon }{d}S,$$

where *ε* is the permittivity, *d* is the thickness, and *S* is the area of the membrane. This applies provided that the ratio *ε*/*d* (representing the specific membrane capacitance) is constant over the entire area of the membrane.

If we consider *ε*/*d* to be a constant in the whole membrane system, the ratio of tubular and surface capacitance *k* = *C*_t_/*C*_s_ equals the ratio of corresponding areas

2 $$\frac{{C_{{\text{t}}} }}{{C_{{\text{s}}} }} = \frac{{S_{{\text{t}}} }}{{S_{{\text{s}}} }} = k.$$

**Fig. 1:**
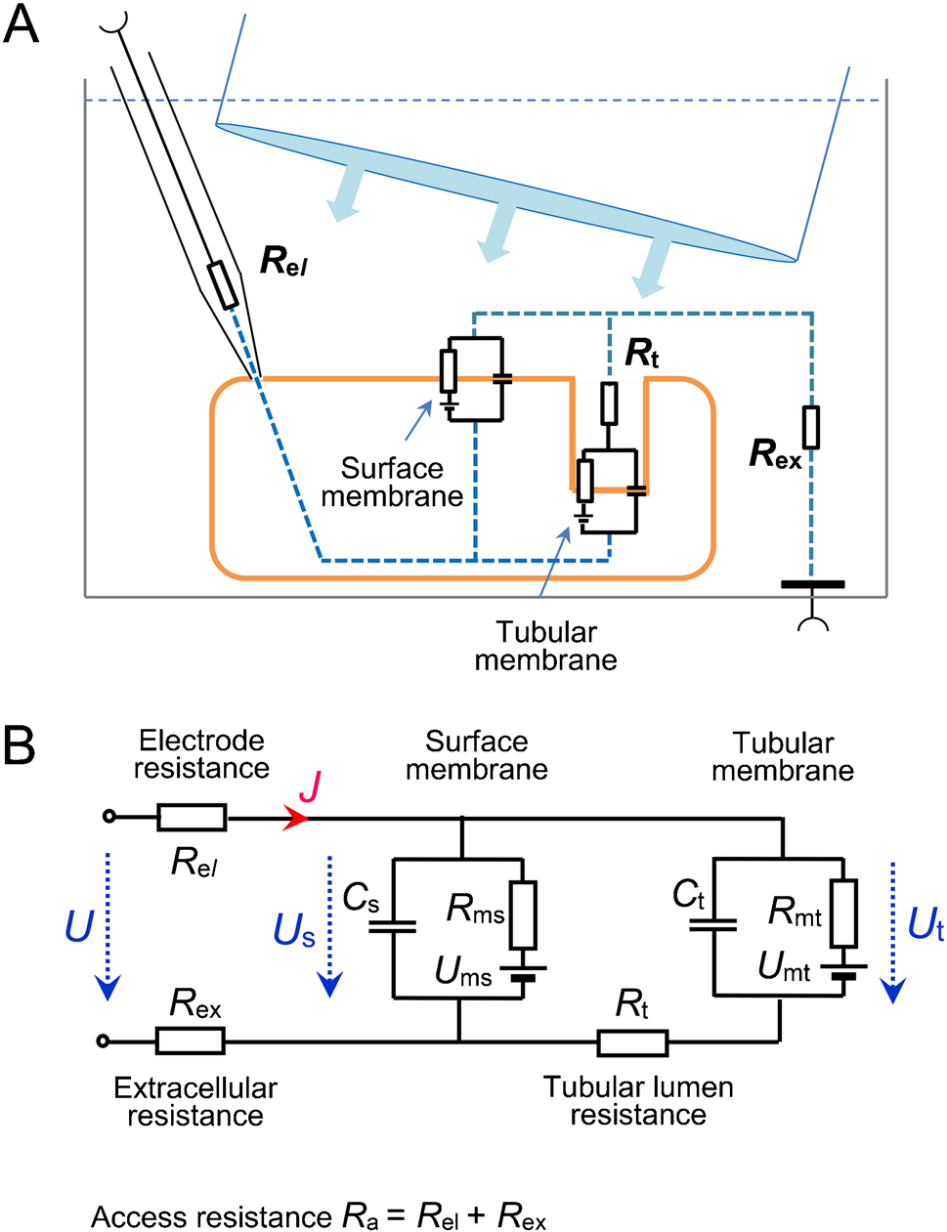
Principle of the method. **A** Experimental setup comprising an isolated cell, a glass microelectrode, and a jet pipe for rapid exchange of extracellular solutions. **B** Lumped-element electrical equivalent circuit of a cell with the developed tubular system connected to the measuring equipment. *R*_el_, glass electrode resistance; *R*_ex_, resistance of the extracellular solution between the ground electrode and the measured cell; *R*_a_, access resistance (*R*_el_ + *R*_ex_) related to the cell as a whole; *C*_s_, *C*_t_, membrane capacitances of the surface and tubular membrane; *R*_ms_, *R*_mt_, membrane resistances of the surface and tubular membrane; *U*_ms_, *U*_mt,_, reversal voltage of the surface and tubular membrane; *R*_t_, resistance of the lumen of the tubular system; *U*, *U*_s_, and *U*_t_, imposed, surface, and tubular membrane voltage, respectively; *J*, membrane current

In cardiomyocytes, simple electrophysiological measurements do not allow the assessment of both capacitances *C*_t_ and *C*_s_ separately because the surface and the tubular systems are tightly electrically coupled. In terms of the model with lumped parameters (Fig. 1), the surface and tubular membranes are separated by the electrical resistance of the lumens of the tubular system *R*_t_. In physiological solution, this resistance is so small that the responses of the membrane current to subthreshold steps of the applied membrane voltage (descending part of the capacitive current) follow a simple exponential waveform (for a detailed analysis, see Appendix 2 of [12]). It follows that only the total membrane capacitance *C*_m_ of the whole membrane system (*C*_m_ = *C*_t_ + *C*_s_) can be estimated from usual electrophysiological measurements and common analysis of the mono-exponential approximation of descending part of the capacitive current.

For the electrophysiological determination of capacitances *C*_s_ and *C*_t_, detubulation methods were developed, consisting in disconnection of tubular membranes by osmotic shock [2, 7, 8, 11]. However, these widely used methods are irreversible, which makes repeated measurements on the same cell and the use of paired difference tests impossible. In addition, the difficult-to-determine fraction of tubules may remain intact after the detubulation procedure [5, 7, 13] which may limit the accuracy of the *C*_t_ and *C*_s_ determination.

The basic idea of the newly proposed method is the electrical separation of the surface and tubular membrane system by increasing the electrical resistance of the tubule lumens. It can be expected that a marked reduction of the electrical coupling between the two membrane systems will transform the virtually mono-exponential course of the recorded capacitive current into two distinguishable exponential components. This justifies the expectation that it will be possible to determine the capacitances *C*_s_ and C_t_ and corresponding ratio *k* (Eq. (2)) from the newly acquired parameters. An increase in the resistance separating the tubular membrane from the surface membrane can be achieved experimentally by a short-term transient replacement of the extracellular solution with the isotonic low-conductive sucrose solution (Fig. 1A). This approach would leave the cell intact and allow repeated measurements.

The determination of the values of the parameters describing the passive electrical properties of the surface and tubular membranes is based on the analysis of the capacitive current recorded in response to the imposed rectangular subthreshold pulses in the sucrose solution. This study includes the derivation of formulas for the calculation of passive parameters of a cell equivalent electrical scheme with lumped parameters, including capacitances of tubular and surface membranes. The derived relationships are verified using a computer model and tested in preliminary experiments. A simplified version of this method limited to the determination of both capacitances was tested in experiments on rat ventricular and atrial cardiomyocytes [17]. The mean value of the tubular membrane fraction ft = Ct/Cm fitted well within the range of values previously obtained by detubulation approaches and was significantly lower in atrial myocytes than in ventricular myocytes. The fraction *f*_t_ and the ratio *k* introduced in (2) are simply related: *f*_t_ = *k*/(*k* + 1). The present study provides a complete theoretical basis and verification of the proposed method. Assuming a direct proportionality between the ratio of membrane conductivities and the ratio of membrane capacitances in tubular and surface membranes, all elements of the electrical equivalent circuit of the measured cardiomyocytes (Fig. 1B) are additionally calculated. The derived formulas are verified by a quantitative model at different optional numerical values of the equivalent circuit.

## Results

### Theoretical background

Figure 1 illustrates a schematic representation of the proposed method for measurement on a cardiomyocyte (Fig. 1A) and a simple electrical equivalent circuit with lumped parameters (Fig. 1B). In the subthreshold range of membrane voltage, the membrane resistances are considered constant, and the electrical equivalent circuit is described mathematically by a system of two non-homogeneous linear differential equations of the first order with respect to time (*t*) for variables *U*_s_ and *U*_t_ representing surface and tubular membrane voltages:

3 $$\frac{{dU_{{\text{t}}} }}{dt} = \frac{1}{{\tau_{{\text{t}}} }}U_{{\text{s}}} - \frac{{k_{{\text{t}}} }}{{\tau_{{\text{t}}} }}U_{{\text{t}}} + \frac{{k_{{{\text{Ut}}}} }}{{\tau_{{\text{t}}} }},\frac{{dU_{{\text{s}}} }}{dt} = - \frac{{k_{{{\text{st}}}} }}{{\tau_{{\text{s}}} }}U_{{\text{s}}} + \frac{{k_{{{\text{at}}}} }}{{\tau_{{\text{s}}} }}U_{{\text{t}}} + \frac{{k_{{{\text{Us}}}} + U}}{{\tau_{{\text{s}}} }},$$

where

4 $$\tau_{{\text{t}}} = R_{{\text{t}}} C_{{\text{t}}} ,\quad \tau_{{\text{s}}} = R_{{\text{a}}} C_{{\text{s}}} ,\quad k_{{\text{t}}} = 1 + \frac{{R_{{\text{t}}} }}{{R_{{{\text{mt}}}} }},\quad k_{{{\text{st}}}} = 1 + \frac{{R_{{\text{a}}} }}{{R_{{{\text{ms}}}} }} + \frac{{R_{{\text{a}}} }}{{R_{{\text{t}}} }},\quad k_{{{\text{at}}}} = \frac{{R_{{\text{a}}} }}{{R_{{\text{t}}} }},\quad k_{{{\text{Ut}}}} = U_{{{\text{mt}}}} \frac{{R_{{\text{t}}} }}{{R_{{{\text{mt}}}} }},\quad k_{{{\text{Us}}}} = U_{{{\text{ms}}}} \frac{{R_{{\text{a}}} }}{{R_{{{\text{ms}}}} }}.$$

$$k_{{{\text{Ut}}}} = U_{{{\text{mt}}}} \frac{{R_{{\text{t}}} }}{{R_{{{\text{mt}}}} }},\quad k_{{{\text{Us}}}} = U_{{{\text{ms}}}} \frac{{R_{{\text{a}}} }}{{R_{{{\text{ms}}}} }}.$$

Equations (3) and (4) can be solved following the standard approach to systems of linear non-homogenous differential equations (e.g., [6]). In the case of the response to the imposed subthreshold step of membrane voltage *U* from the level *U*_1_ to *U*_2_, the solution of Eqs. (3) and (4) leads to a sum of two exponential functions

5 $$U_{{\text{s}}} (t) = c_{1} {\kern 1pt} e^{{ - \frac{t}{{\tau_{1} }}}} + c_{2} {\kern 1pt} e^{{ - \frac{t}{{\tau_{2} }}}} + \frac{{k_{{\text{t}}} {\kern 1pt} k_{{{\text{Us}}}} + k_{{{\text{Ut}}}} + k_{{\text{t}}} {\kern 1pt} U_{2} }}{{k_{{{\text{st}}}} {\kern 1pt} k_{{\text{t}}} - k_{{{\text{at}}}} }},$$

6 $$U_{{\text{t}}} (t) = \frac{{c_{1} }}{{k_{{\text{t}}} - \frac{{\tau_{{\text{t}}} }}{{\tau_{1} }}}}{\kern 1pt} \,e^{{ - \frac{t}{{\tau_{1} }}}} + \frac{{c_{2} }}{{k_{{\text{t}}} - \frac{{\tau_{{\text{t}}} }}{{\tau_{2} }}}}{\kern 1pt} \,e^{{ - \frac{t}{{\tau_{2} }}}} + \frac{{k_{{{\text{Us}}}} + k_{{{\text{Ut}}}} {\kern 1pt} k_{{{\text{Us}}}} + {\kern 1pt} {\kern 1pt} U_{2} }}{{k_{{{\text{st}}}} {\kern 1pt} k_{{\text{t}}} - k_{{{\text{at}}}} }}.$$

Considering initial conditions, the constants *c*_1_ and *c*_2_ are expressed as

7 $$c_{1} = \frac{{U_{1} - U_{2} }}{{k_{{{\text{st}}}} {\kern 1pt} k_{{\text{t}}} - k_{{{\text{at}}}} }}{\kern 1pt} \,\frac{{\tau_{1} {\kern 1pt} k_{{\text{t}}} - \tau_{{\text{t}}} }}{{\tau_{1} {\kern 1pt} - \tau_{2} {\kern 1pt} }},\quad \quad c_{2} = \frac{{U_{1} - U_{2} }}{{k_{{{\text{st}}}} {\kern 1pt} k_{{\text{t}}} - k_{{{\text{at}}}} }}{\kern 1pt} \,\frac{{\tau_{{\text{t}}} - \tau_{2} {\kern 1pt} k_{{\text{t}}} }}{{\tau_{1} {\kern 1pt} - \tau_{2} {\kern 1pt} }}.$$

The time constants *τ*_1_ and *τ*_2_ of the two exponential terms satisfy the conditions arising from the properties of the roots of the characteristic equation

8 $$\frac{{k_{{\text{t}}} }}{{\tau_{{\text{t}}} }} + \frac{{k_{{{\text{st}}}} }}{{\tau_{{\text{s}}} }} = \frac{1}{{\tau_{1} {\kern 1pt} }} + \frac{1}{{\tau_{2} {\kern 1pt} }},$$

9 $$\frac{{k_{{{\text{st}}}} {\kern 1pt} k_{{\text{t}}} - k_{{{\text{at}}}} }}{{\tau_{{\text{s}}} \tau_{{\text{t}}} }} = \frac{1}{{\tau_{1} {\kern 1pt} \tau_{2} }}.$$

The only measured quantity is membrane current *J,* which is simply related to the membrane voltage *U*_s_ by Ohm’s law

10 $$J = \frac{{U - U_{{\text{s}}} }}{{R_{{\text{a}}} }}.$$

To express the response of the current *J* to a small step of the membrane voltage (from *U*_1_ to *U*_2_), it is necessary to substitute *U* = *U*_2_ and *U*_s_ from Eq. (5) into (10). Obviously, the current *J* can be described by a sum of two exponential terms and a constant

11 $$J = J_{1} {\kern 1pt} e^{{ - \frac{t}{{\tau_{1} }}}} + J_{2} {\kern 1pt} e^{{ - \frac{t}{{\tau_{2} }}}} + J_{\infty ,2} .$$

Numerical values of the magnitudes (*J*_1_, *J*_2_), corresponding time constants (*τ*_1_, *τ*_2_) of both components, and steady current level (*J*_∞,2_) can be determined by bi-exponential approximation of the recorded current response. After supplementing the steady current level (*J*_∞,1_) at the voltage *U*_1_, we obtain numerical values of six parameters *J*_1_, *J*_2_, *J*_∞,1_, *J*_∞,2_, *τ*_1_, and *τ*_2_, from which the values of the important parameters of the electrical equivalent scheme (Fig. 1B) can be expressed. Substituting from Eqs. (5)–(9) into (10), we get

12 $$J_{1} = \frac{{U_{2} - U_{1} }}{{R_{{\text{a}}} }}\frac{1}{{k_{{{\text{st}}}} {\kern 1pt} k_{{\text{t}}} - k_{{{\text{at}}}} }}{\kern 1pt} \,\frac{{\tau_{1} {\kern 1pt} k_{{\text{t}}} - \tau_{{\text{t}}} }}{{\tau_{1} {\kern 1pt} - \tau_{2} {\kern 1pt} }},$$

13 $$J_{2} = \frac{{U_{2} - U_{1} }}{{R_{{\text{a}}} }}\frac{1}{{k_{{{\text{st}}}} {\kern 1pt} k_{{\text{t}}} - k_{{{\text{at}}}} }}{\kern 1pt} \,\frac{{\tau_{{\text{t}}} - \tau_{2} {\kern 1pt} k_{{\text{t}}} }}{{\tau_{1} {\kern 1pt} - \tau_{2} {\kern 1pt} }},$$

14 $$J_{\infty ,1} = \frac{{U_{1} \left( {k_{{{\text{st}}}} {\kern 1pt} k_{{\text{t}}} - k_{{{\text{at}}}} - k_{{\text{t}}} } \right) - k_{{\text{t}}} {\kern 1pt} k_{{{\text{Us}}}} - k_{{{\text{at}}}} {\kern 1pt} k_{{{\text{Ut}}}} }}{{R_{{\text{a}}} {\kern 1pt} \left( {k_{{{\text{st}}}} {\kern 1pt} k_{{\text{t}}} - k_{{{\text{at}}}} } \right)}},$$

15 $$J_{\infty ,2} = \frac{{U_{2} \left( {k_{{{\text{st}}}} {\kern 1pt} k_{{\text{t}}} - k_{{{\text{at}}}} - k_{{\text{t}}} } \right) - k_{{\text{t}}} {\kern 1pt} k_{{{\text{Us}}}} - k_{{{\text{at}}}} {\kern 1pt} k_{{{\text{Ut}}}} }}{{R_{{\text{a}}} {\kern 1pt} \left( {k_{{{\text{st}}}} {\kern 1pt} k_{{\text{t}}} - k_{{{\text{at}}}} } \right)}}.$$

### Elements of the electrical equivalent circuit

The numerical values of the six parameters *J*_1_, *J*_*2*_, *J*_∞,1_, *J*_*∞,2*_, *τ*_1_, and *τ*_2_ resulting from the approximation of the recorded capacitive current by the bi-exponential function (11) can be entered into the six derived equations ((8), (9), (12), (13), (14), and (15)). However, only a limited number of the eight elements forming the equivalent circuit in Fig. 1B can be calculated from these equations.

The access resistance *R*_a_ and the capacitance of the surface membrane *C*_s_ could be expressed from Eqs. (4), (8), (9), and (12)–(15) after rearrangements:

16 $$R_{{\text{a}}} = \frac{{U_{2} - U_{1} }}{{J_{1} + J_{2} - J_{\infty ,1} + J_{\infty ,2} }},$$

17 $$C_{{\text{s}}} = \frac{{\tau_{{\text{s}}} }}{{R_{{\text{a}}} }}\,,{\text{ where }}\tau_{{\text{s}}} = \left( {J_{1} + J_{2} - J_{\infty ,1} + J_{\infty ,2} } \right)\;\frac{{\tau_{1} \tau_{2} }}{{\tau_{1} J_{2} + \tau_{2} J_{1} }}\,.$$

The resistances of tubular membrane and tubular lumen could not be calculated directly. However, two combinations of resistances *R*_ms_, *R*_mt_, and *R*_t_ (denoted *R*_1_ and *R*_2_) could be calculated as.

18 $$\begin{aligned}R_{1}& = R_{{{\text{ms}}}} ||R_{{\text{t}}} = \frac{{R_{{{\text{ms}}}} R_{{\text{t}}} }}{{R_{{{\text{ms}}}} + R_{{\text{t}}} }} = \frac{{R_{{\text{a}}} }}{b - 1},\\ &\quad{\text{where}} \, b = \frac{{\tau_{1}^{2} J_{2} + \tau_{2}^{2} J_{1} }}{{\tau_{1} J_{2} + \tau_{2} J_{1} }}\;\frac{{\tau_{{\text{s}}} }}{{\tau_{1} \tau_{2} }},\end{aligned}$$

19 $$\begin{aligned}R_{2}& = R_{{{\text{ms}}}} ||(R_{{\text{t}}} + R_{{{\text{mt}}}} ) = \frac{{R_{{{\text{ms}}}} (R_{{\text{t}}} + R_{{{\text{mt}}}} )}}{{R_{{{\text{ms}}}} + R_{{\text{t}}} + R_{{{\text{mt}}}} }} = R_{{\text{a}}} \frac{a}{1 - a},\\ &\quad{\text{ where }}a = R_{{\text{a}}} \frac{{J_{1} + J_{2} }}{{U_{2} - U_{1} }}.\end{aligned}$$

For convenience, parallel combinations of resistances are expressed by the symbol ||. This notation will be retained in the whole text.

The tubular membrane capacitance *C*_t_ could theoretically be expressed as

20 $$C_{{\text{t}}} = \frac{{\tau_{1} J_{2} + \tau_{2} J_{1} }}{{J_{2} + J_{1} }}\frac{1}{{R_{{{\text{mt}}}} ||R_{{\text{t}}} }}.$$

However, Eqs. (8), (9), and (12)–(15) did not allow to express formula for the calculation of parallel combination *R*_mt_ || *R*_t_. We looked at two ways to solve this problem. One possibility was the substitution of parallel combination *R*_mt_ || *R*_t_ for *R*_ms_ || *R*_t_, which was justified because *R*_mt_ >  > *R*_t_ and *R*_ms_ >  > *R*_t_ (as directly confirmed by calculating the resistances *R*_ms_, *R*_mt_, and *R*_t_ in the present study). This procedure was recently verified in experiments on rat ventricular and atrial cardiomyocytes [17]. The above substitution led to the calculation formula

$$C_{{\text{t}}} \sim \frac{{\tau_{1} J_{2} + \tau_{2} J_{1} }}{{J_{{1}} + J_{{2}} }}\frac{{k_{c} }}{{R_{1} }},$$

where the coefficient *k*_c_ (0.97 for ventricular and to 0.91 for atrial cardiomyocytes) was introduced as a correction for the mean error caused by the exchange of membrane resistances *R*_mt_ for *R*_ms_ in the approximate calculation of *C*_t_ as justified in [17].

Here, we describe another possibility consisting in the introduction of an additional presumption instead of the simplification introduced in the experimental study [17]. This approach is more general and allows the calculation of the membrane resistances *R*_ms_ and *R*_mt_ and the tubular resistance *R*_t_ in addition to capacitances. It is reasonable to expect the ratio of membrane conductance *G*_mt_ /*G*_ms_ (= *R*_ms_ /*R*_mt_) to be proportional to the ratio of membrane areas like the ratio *C*_t_ /*C*_s_ according to Eq. (2):

21 $$\frac{{G_{{{\text{mt}}}} }}{{G_{{{\text{ms}}}} }} = \frac{{R_{{{\text{ms}}}} }}{{R_{{{\text{mt}}}} }} = \gamma \frac{{S_{{\text{t}}} }}{{S_{{\text{s}}} }} = \gamma \frac{{C_{{\text{t}}} }}{{C_{{\text{s}}} }} = \gamma k,$$

where *ɣ* is a hitherto unknown coefficient of proportionality, the value of which may be different from 1 regarding the heterogeneity of tubular membrane (namely due to the differences in the distribution of ionic channels). From Eqs. (12), (13), and (15), the quadratic equation for *R*_ms_ as a function of *ɣk* can be derived

22 $$R_{{{\text{ms}}}}^{2} - R_{{{\text{ms}}}} \left( {R_{1} + R_{2} + \left( {R_{2} - R_{1} } \right)\gamma k} \right) + R_{2} R_{1} = 0.$$

Only one root of the Eq. (22)

23 $$\begin{aligned}R_{{{\text{ms}}}}& = 0.5(R_{1} + R_{2} + (R_{2} - R_{1} )\gamma k) \\ &\quad+0.5 ((R_{1} + R_{2} + (R_{2} - R_{1} )\gamma k)^{2} - 4R_{2} R_{1} )^{0.5} )\end{aligned}$$

leads to a physically realistic solution. The resistances *R*_1_ and *R*_2_ are directly computable from Eqs. (18) and (19).

By inserting expressions of *C*_t_ and *C*_s_ (Eqs. (20) and (17)) into Eq. (2), and considering the definition (18) of *R*_1_, we get another expression of *R*_ms_ as a function of *k* and *ɣ*

24 $$R_{{{\text{ms}}}} = \frac{{(\gamma k - 1)R_{1} R_{1,2} }}{{k{\kern 1pt} R_{1} - R_{1,2} }},{\text{ where }}R_{1,2} = \frac{{R_{{\text{a}}} }}{{\tau_{{\text{s}}} }}\frac{{\tau_{1} J_{2} + \tau_{2} J_{1} }}{{J_{2} + J_{1} }}$$

The numeric values of the variables *R*_ms_ and *k* can be calculated (for a selected *ɣ* value) from the system of two Eqs. (23) and (24). The constant *k* can also be calculated from one implicit equation after comparing the right sides of the Eqs. (23) and (24).

The next section will show how the calculated value of *k* and the membrane capacitances (*C*_t_, *C*_s_) depend on the *ɣ* setting. All the constants in  Eqs. (23) and (24) can be calculated from the parameters *J*_1_, *J*_2_, *J*_∞,1_, *J*_∞,2_, *τ*_1_, and *τ*_2_ determined from the results of fitting the capacitive current response (to a small voltage step) by a sum of two exponential functions and a constant. Calculation of the constant *k* makes it possible to quantify other elements of the electrical equivalent circuit (Fig. 1B). In addition to the expressions derived so far for *R*_a_, *C*_s_, and *R*_ms_ (Eqs. (16), (17), and (24)), the remaining elements can be calculated as follows: the most important parameter *C*_t_ follows from Eq. (2)

25 $$C_{{\text{t}}} = k\,C_{{\text{s}}} ,$$

the resistances *R*_t_ and *R*_mt_ result from Eqs. (18) and (21)

26 $$R_{{\text{t}}} = \frac{{R_{1} R_{{{\text{ms}}}} }}{{R_{{{\text{ms}}}} - R_{1} }}, \quad\, R_{{{\text{mt}}}} = \frac{{R_{{{\text{ms}}}} }}{\gamma k}.$$

The total membrane capacitance and the fraction of tubular capacitance can be expressed as

27 $$C_{{\text{m}}} = C_{{\text{s}}} + C_{{\text{t}}} {\kern 1pt} ,\quad \,\quad f_{{\text{t}}} = \frac{{C_{{\text{t}}} }}{{C_{{\text{s}}} + C_{{\text{t}}} }} = \frac{k}{1 + k} = \frac{{S_{{\text{t}}} }}{{S_{{\text{s}}} + S_{{\text{t}}} }}{\kern 1pt} .$$

The formulas allowing quantification of the elements of the electrical equivalent circuit are summarized in Table 1.

**Table 1:** Mathematical formulas for calculation of the elements of electrical equivalent circuit

Basic parameters					
***R***_**a**_	***C***_**s**_	***C***_**t**_	***R***_**ms**_	***R***_**mt**_	***R***_**t**_
$\frac{U_2-{U}_1}{J_1+{J}_2-{J}_{\infty, 1}+{J}_{\infty, 2}}$	$\frac{\tau_s}{R_a}$	*k C*_*s*_	$\frac{\left(\gamma k-1\right){R}_1{R}_{1,2}}{kR_1-{R}_{1,2}}$	$\frac{R_{\textrm{ms}}}{k}$	$\frac{R_1{R}_{\textrm{ms}}}{R_{\textrm{ms}}-{R}_1}$
Auxiliary quantities					
***τ***_**s**_		***R***_**1,2**_		***b***	
$\frac{J_1+{J}_2-{J}_{\infty, 1}+{J}_{\infty, 2}}{\tau_1{J}_2+{\tau}_2{J}_1}{\tau}_1{\tau}_2$		$\frac{R_a{\tau}_1{J}_2+{\tau}_2{J}_1}{\tau_{\textrm{s}}\ {J}_2+{J}_1}$		$\frac{\tau_1^2{J}_2+{\tau}_2^2{J}_1}{\tau_1{J}_2+{\tau}_2{J}_1}\frac{\tau_s}{\tau_1{\tau}_2}$	
***R***_**1**_		***a***		***R***_**2**_	
$\frac{R_a}{b-1}$		$\frac{J_1+{J}_2}{J_1+{J}_2-{J}_{\infty, 1}+{J}_{\infty, 2}}$		${R}_a\frac{a}{1-a}$	

^*^Calculation of the elements of electrical equivalent circuit (Fig. 1B) from the parameters *J*_1_, *J*_2_, *J*_∞,1_, *J*_∞,2_, *τ*_1_ and *τ*_2_ resulted from a double-exponential analysis of current response to a subthreshold step of membrane voltage. The value of *k* results from the solution of the Eqs. (23) and (24)

The values of the reversal voltages *U*_ms_ and *U*_mt_ can be estimated from the parameters *J*_1_, *J*_2_, *J*_∞,1_, *J*_∞,2_, *τ*_1_, and *τ*_2_ only approximately under the assumption that *U*_ms_ = *U*_mt_ (which may not be exactly met): the relations *U*_ms_ ≈ *U*_mt_ ≈ *U*_1_ – *J*_∞,1_ *R*_a_ /(1-*a*) = *U*_2_ – *J*_∞,2_ *R*_a_ /(1-*a*) follow from Eqs. (14, 15). However, the calculated values of *C*_t_, *C*_s_, and *f*_t_ are independent of the values of *U*_ms_ and *U*_mt_ used for calculations.

### Model verification of the theory

To prove the correctness of the described calculations of the elements of the electrical equivalent circuit, we designed software written in MATLAB Live Editor (S1_verification.mlx available on request from the corresponding author), which is based on the solution of the set of differential Eqs. (3, 4). The software was designed to mimic real experiments on isolated cells. The numerical values of *R*_a_, *R*_t_, *R*_ms_, *R*_mt_, *C*_s_, *C*_t_, *U*_ms_, and *U*_mt_ are optional. The values summarized in Table 2 were chosen as examples of values close to those obtained from preliminary experiments. The voltage levels *U*_1_ and *U*_2_ were set to – 80 and − 75 mV. However, the calculated values of the elements of the electrical equivalent circuit do not depend on this choice. The results obtained by the calculations according to the derived relations are then compared to the selected parameter values of the cell equivalent scheme (Table 2) to verify the theory.

**Table 2:** Values of the elements of electrical equivalent circuit used for verification of the derived formulas

*R*_a [MΩ]_	*R*_t [MΩ]_	*R*_ms [MΩ]_	*R*_mt [MΩ]_	*C*_s [pF]_	*C*_t [pF]_	*U*_ms [mV]_	*U*_mt [mV]_
12.5	15	150	241	74	46	− 160	− 160

**Fig. 2:**
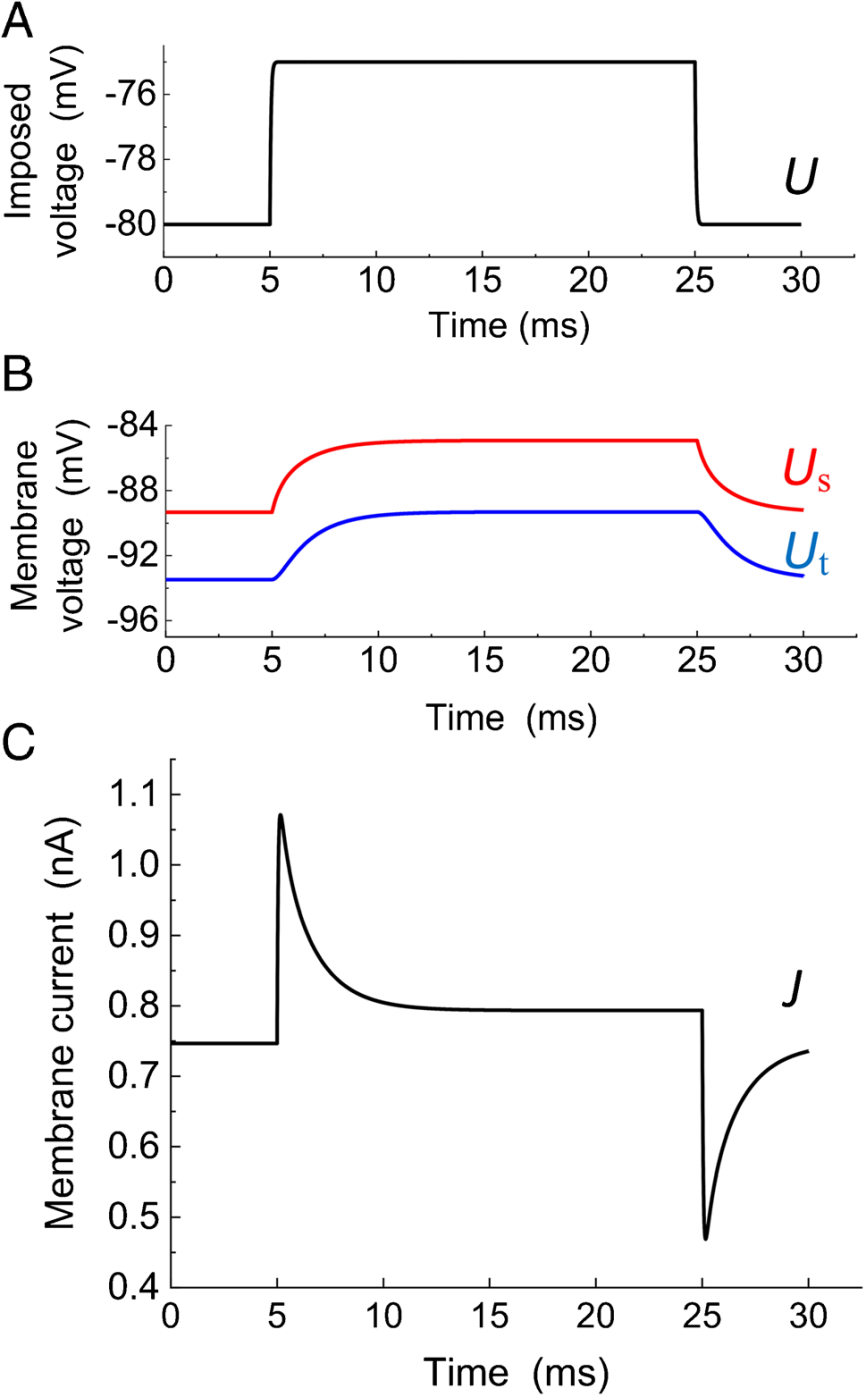
Solution of the equations describing the electrical equivalent circuit shown in Fig. 1B. Equations (3 and (4) were solved with parameter values given in Table 2. **A** Imposed voltage impulse *U*; an additional simple differential equation was simultaneously solved to create a slower rising and falling edge of the rectangular voltage impulse closer to actual shape. **B** Responses of surface and tubular membrane voltage *U*_s_ and *U*_t_. **C** Response of the membrane current *J*

The shape of the imposed rectangular voltage impulse mimicking experimental records with limited rising and falling edges (Fig. 2A) resulted from simultaneously solving an additional simple differential equation to create fast exponential onset and offset of the imposed impulses with the optional time constant (*τ*_p_ = 0.05 ms was used in most computations). Figure 2B shows computed responses of surface and tubular membrane voltage (*U*_s_ and *U*_t_). The characteristics of the experimental capacitive current with a steep increase to a maximum followed by a slow decay are reproduced in the simulated current response (Fig. 2C).

**Fig. 3:**
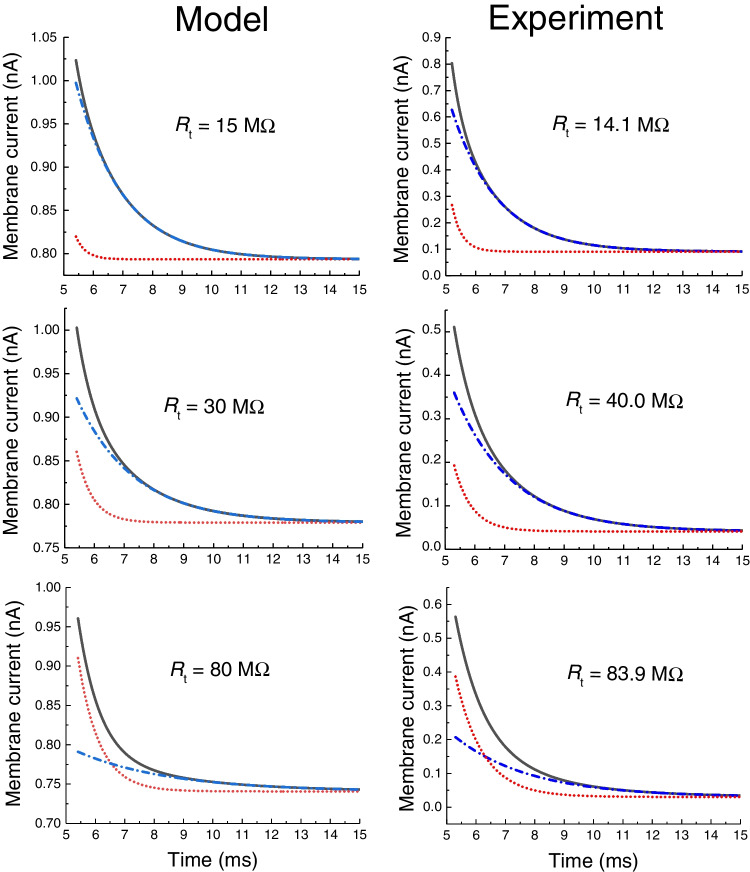
Comparison of the bi-exponential approximation of the calculated and recorded descending phase of capacitive current. The evaluation started with some delay after the onset of the depolarization step. Model: calculated currents resulting from the solution of differential Eqs. (3, 4) with values of parameters according to Table 2 (except for the variable values of the resistance *R*_t_ marked in the graphs). The calculated currents are indistinguishable from their bi-exponential approximations (overlaid black solid lines). Both components of the bi-exponential approximation are marked out as dashed and dotted (blue) and dotted (red) lines. Experiment: results of analysis from three selected rat ventricular cells

The descending phase of the simulated capacitive current was expected to follow a distinct bi-exponential course since the tubular resistance *R*_t_ was set to a sufficiently high value corresponding to the effect of sucrose solution. Using the *Curve fitting tool* of MATLAB (R 2017a), the descending phase of capacitive current was indeed well fitted by the bi-exponential function (left column in Fig. 3). Three different *R*_t_ values were selected (15, 30, and 80 MΩ) within the range observed in experiments. Similarly, three cells from a set of experimental results were selected and analyzed for comparison (right column in Fig. 3). As apparent, the data from the model and the experiment matched well, although other elements of the equivalent scheme beside *R*_t_ affect the course of the capacitive current.

The decomposition of the descending phase of the capacitive current determines the numerical values of the five parameters *J*_1_, *J*_2_, *J*_∞,2_, *τ*_1_, and *τ*_2_. After supplementing with the steady state current *J*_*∞,*1_ read at the holding voltage, the six parameters were inserted into Eqs. (16)–(19) and (23)–(27) to calculate the elements of the electrical equivalent scheme, which are then compared with the values set in Table 2.

**Fig. 4:**
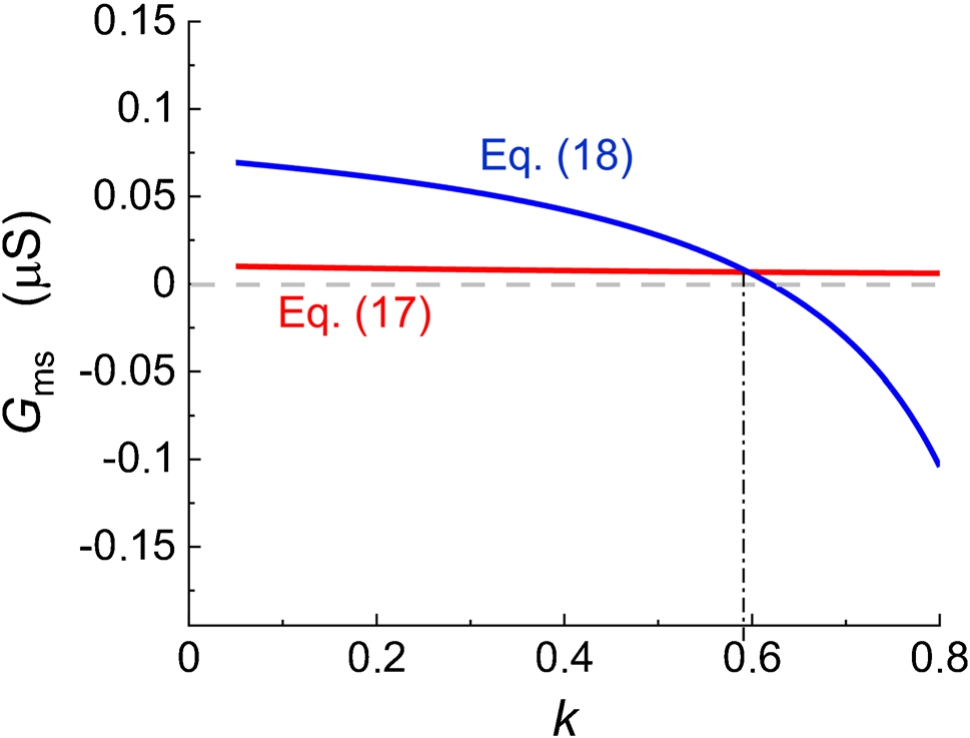
Determination of the principal parameters. The surface membrane conductivity *G*_ms_ was calculated in Matlab as a function of *k* from Eq. (23) (red lines) and from Eq. (24) (blue lines). The real values of *k* and *G*_ms_ were read at the point of intersection of the two curves

The next key point was the estimation of the surface membrane conductance *G*_ms_ = 1/*R*_ms_ and the constant of proportionality *k* between the tubular and surface membrane capacitance. The value of the constant *ɣ* related to the *G*_mt_ /*G*_ms_ ratio (21) was still unknown. To verify the derived formulas, we first solved the system of two Eqs. (23) and (24) assuming *ɣ* = (*G*_mt_/*G*_ms_) (*C*_s_/*C*_t_) to satisfy exactly Eq. (21). The choice of parameters according to Table 2 resulted in *ɣ* ~ 1. As shown in Fig. 4 illustrating the graphical solution of the Eqs. (23) and (24), the numerical values of *G*_ms_ and *k* are given by the intersection of the two plotted curves.

**Fig. 5:**
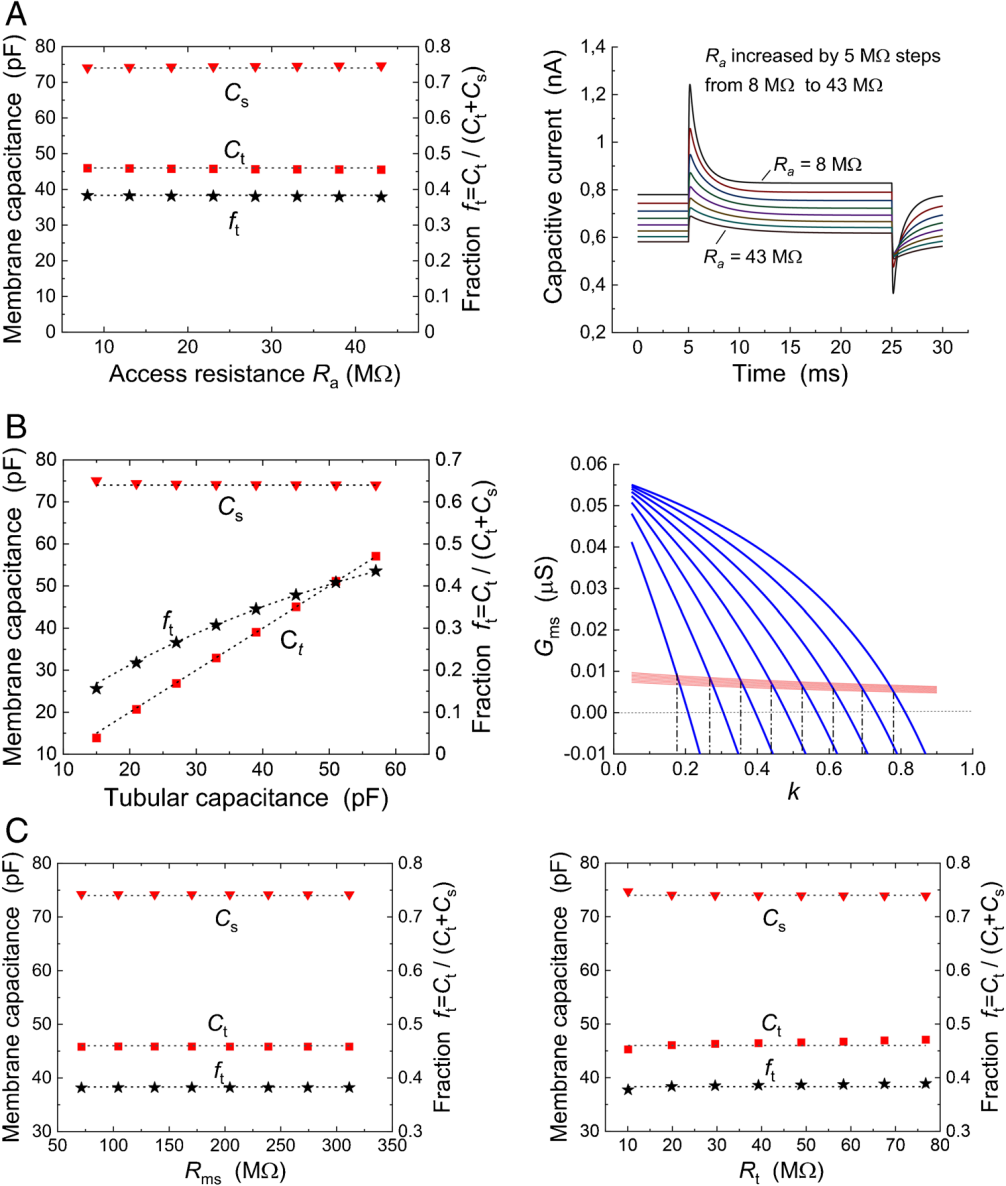
Verification of the proposed approach to determine the membrane capacitances *C*_s_, *C*_t_, and the fraction of tubular capacitance/area *f*_t_. The results of the calculations demonstrate insensitivity of the quantities *C*_s_, *C*_t_, and *f*_t_ to: **A** variations in access resistance *R*_a_ (right panel shows time courses of the capacitive current), **B** variations in tubular capacitance *C*_t_ (right panel illustrates the determination of *k* = *C*_t_ /*C*_s_ from Eqs. (23) and (24), **C** variations in surface membrane resistance *R*_ms_ (left) and resistance of the tubular lumen *R*_t_ (right). Filled symbols: calculated values of *C*_s_, *C*_t_ = *k C*_s_ and *f*_t_ = *k*/(1 + *k*); dotted lines: preselected values of *C*_s_ = 74 pF and *C*_t_ = 46 pF in **A** and **C** or preselected variation of *C*_t_ and thus *f*_t_ in **B**

The main objective of the proposed method was the evaluation of the membrane capacitances *C*_t_ and *C*_s_ and the fraction of tubular capacitance *f*_t_ = *C*_t_ /(*C*_t_ + *C*_s_) as an estimate of the fraction of tubular membrane area *S*_t_ /(*S*_t_ + *S*_s_). Hence, it was necessary to prove that these quantities calculated applying the proposed approach were independent of the values of other elements of the electrical equivalent circuit (Fig. 5). In real experiments on cardiac cells, the value of *γ* satisfying Eq. (21) will be referred to as the “true *γ-*value.” It is unknown in advance. However, to calculate the elements of the electrical equivalent scheme, an estimate of *γ* is required which will be referred to as the “expected *γ*-value.” It is important to estimate the error caused by the difference between the expected and the true *γ*-values which will later be explored in the range between 0.4 and 1.25. Let us now set the expected *γ-*value in the middle of this range to a value of 0.7 while the resistance of the tubular membrane *R*_mt_ will be variable to satisfy Eq. (15). This required setting *R*_mt_ = (*R*_ms_/*ɣ*) (C_s_/*C*_t_).

First, we investigated the effect of changes in the access resistance *R*_a_ while maintaining the values of other parameters (except for the variable *R*_mt_) according to Table 2 (Fig. 5A, left).

The pre-set values of *C*_s_, *C*_t_, and *f*_t_ (dotted lines) were well reproduced by calculations applying the derived equations (filled symbols) despite marked variations in the time courses of capacitive current (right). The correctness of the derived formulas was confirmed also at variable tubular membrane capacitance *C*_t_ settings (Fig. 5B, left). The right panel illustrates the assessment of the coefficient *k* (in the way shown in Fig. 4); *k* changed with changes in *C*_t_ while *C*_s_ remained constant. Similarly, the capacitance values were well reproduced when the resistances *R*_ms_ (Fig. 5C, left) and *R*_t_ (Fig. 5C, right) were altered. As stated above, in all these calculations, the condition determining the interdependence of membrane resistances *R*_ms_ and *R*_mt_ Eq. (21) was presumed to be met (illustrated for *γ* = 0.7). The error due to inaccuracy in the fitting procedure did not exceed 1%.

**Fig. 6:**
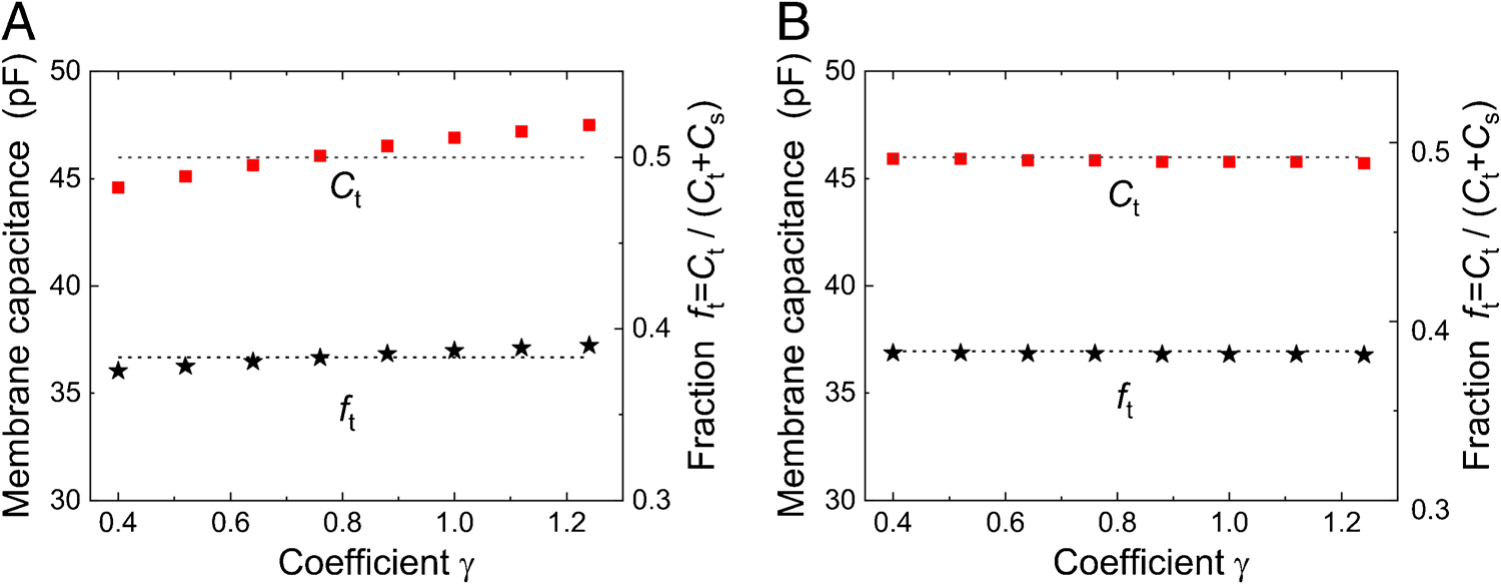
Estimation of the error in *C*_t_ and *f*_t_ determination caused by a difference between the expected *γ*-value and the true *γ-*value (satisfying condition (21))**.** The expected *γ-*values ranged between 0.4 and 1.25. The calculated *C*_s_ (not shown) did not depend on the *γ-*value and was not subject to error. **A** Values of all parameters were set according to Table 2 (except for *R*_mt_ = 345 MΩ adjusting the pre-set *γ*-value to 0.7). The error in the evaluation of *C*_t_ and *f*_t_ did not exceed 4% over the entire range of expected *γ*-values. Note the zero error if the expected *γ* = 0.7. **B** For comparison, the resistance of the tubular membrane was set to *R*_mt_ = (*R*_ms_ /*ɣ*) (C_s_/*C*_t_) so that the condition of Eq. (21) has always been met. Dotted lines—preselected values of *C*_t_ and *f*_t_

The error due to the difference between the expected and the true *γ*-value is evaluated in Fig. 6. The tubular capacitance *C*_t_ and the fraction *f*_t_ are plotted as a function of the expected *γ*-value in the range between 0.4 and 1.25 while the true *γ*-value remained at 0.7. This was achieved by keeping the values of all parameters setting according to Table 2 except for the change of *R*_mt_ to 345 MΩ. The calculated *C*_s_ does not depend on the *γ-*value and is not subject to error. The error in the evaluation of *C*_t_ and *f*_t_ did not exceed 4% in the whole range of expected *γ*-values (Fig. 6A). For comparison, Fig. 6B shows that the error became negligible when the resistance of the tubular membrane was set to *R*_mt_ = (*R*_ms_/*ɣ*) (C_s_/*C*_t_), so that the condition of Eq. (21) was permanently satisfied.

### Use of the method in experiments on ventricular cardiomyocytes

**Fig. 7:**
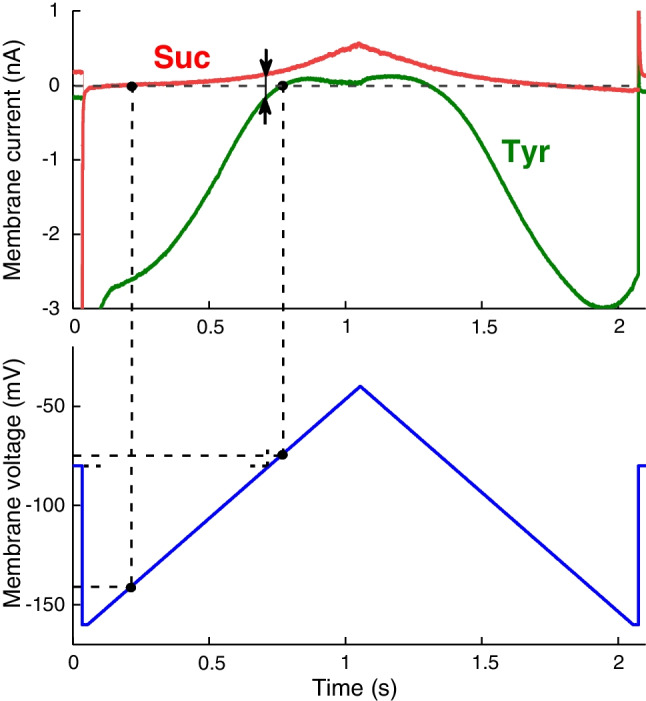
Comparison of quasi steady-state current–voltage relationship in isotonic sucrose (Suc) and Tyrode (Tyr) solution. Membrane currents (top panel) were recorded in response to changes in the membrane voltage composed of 1 s ascending and 1 s descending ramp function in the range between − 160 and − 40 mV (bottom panel). Note that the resting membrane voltage in Tyrode solution (− 74 mV) was shifted to approximately − 140 mV in sucrose solution (dashed lines). When Tyrode solution was replaced with sucrose solution, the inward current at the holding voltage of − 80 mV reversed into an outward current (arrows)

To investigate changes in the membrane current caused by exposure to isotonic sucrose solution, a 2 s ramp membrane voltage from − 160 to − 40 mV and back at 0.1 Hz was applied to the enzymatically isolated rat cardiomyocyte (Fig. 7, bottom panel). When Tyrode solution was replaced with sucrose solution, the inward current at a holding voltage of − 80 mV was reversed (Fig. 7, top panel). The reversal membrane voltage, which was approximately − 75 mV in Tyrode solution, was shifted to around − 140 mV in sucrose solution. The recorded current probably corresponded mainly to the potassium current *I*_K1_ as discussed later.

The newly developed method was tested in a pilot set of experiments on rat ventricular myocytes (*n* = 20). A train of 300 rectangular voltage steps (20 ms, 10 or 5 mV from the holding voltage of − 80 mV) was applied at 25 Hz to reach the steady state. The last 50 current responses were averaged and evaluated. This procedure was repeatedly applied in the sucrose and Tyrode solution.

It was important to find out to what extent the resulting values of the main parameters depended on the estimate of the *γ* coefficient in experiments. The surface membrane capacitance *C*_s_ calculated from Eqs. (16) and (17) did not depend on *γ* and reached value of 92.0 ± 5.4 pF. The tubular membrane capacitance *C*_t_ and the fraction of tubular membrane *f*_t_ amounted 45.7 ± 4.3 pF and 0.327 ± 0.018, respectively. These values obtained at *γ* = 1.2 were closest to the values obtained in our previously published study where the estimated *C*_s_, *C*_t_, and *f*_t_ were 92.7 ± 5.9 pF, 47.3 ± 3.9 pF, and 0.337 ± 0.017, respectively (for details, see ref. [17]). The key parameter *f*_t_ decreased slightly if calculated at *γ* = 0.7, but the difference was only about 3%.

The newly obtained values of membrane and tubular resistances in sucrose solution (mean ± SE) calculated according to Eqs. (24) and (26) from available data (20 cells) were *R*_mt_ = 518.6 ± 79.3 MΩ, *R*_ms_ = 263.8 ± 30.3 MΩ, and *R*_t_ = 30.2 ± 3.4 MΩ. The rough estimate of reversal voltages (assuming *U*_s_ = *U*_t_) was *U*_ms_ ≈ *U*_mt_ ≈ − 149.6 ± 5.2 mV.

**Fig. 8:**
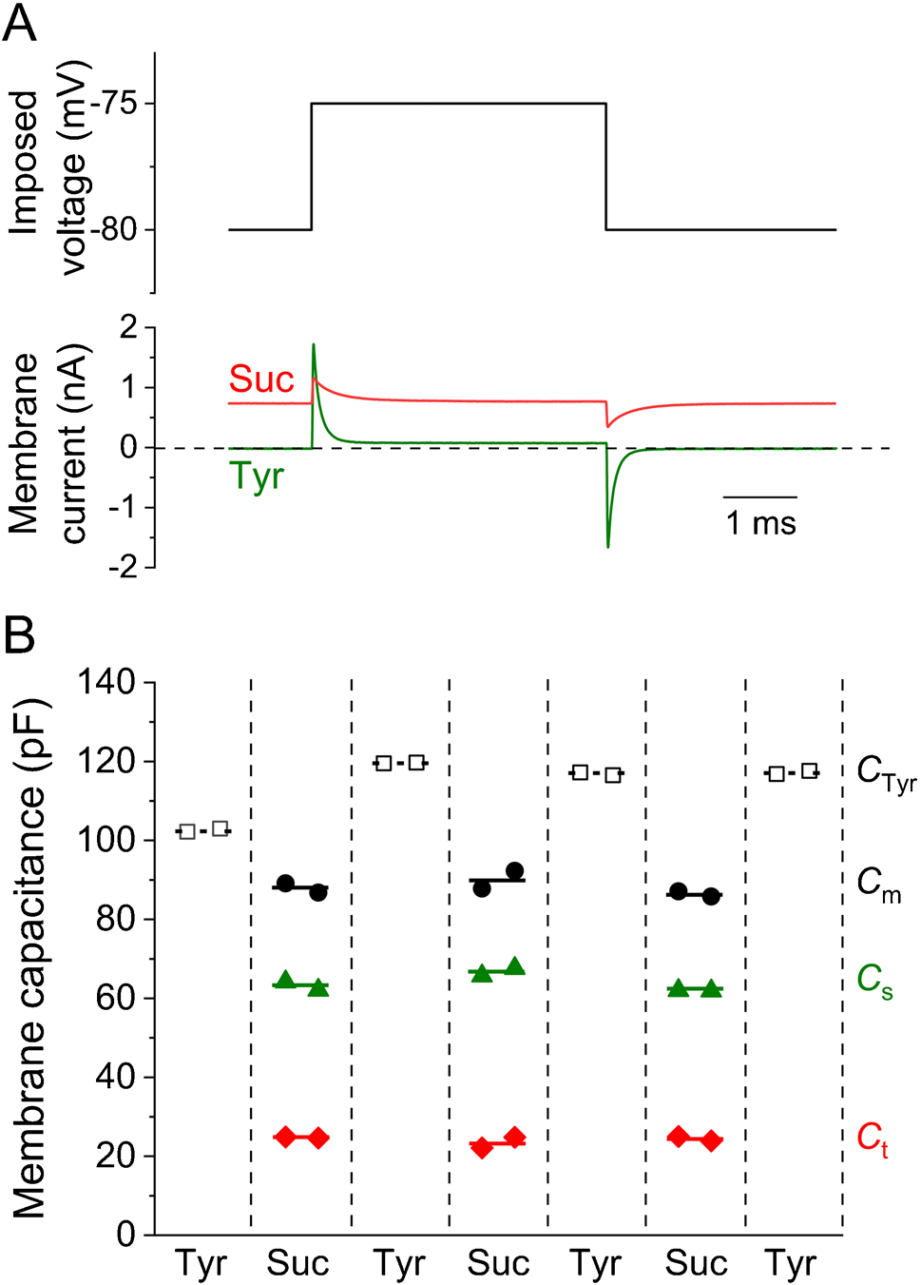
Experimental analysis of tubular and surface membrane capacitances using the newly developed method. **A** Scheme of the experimental protocol (upper panel) and representative recordings of the membrane current in Tyrode (Tyr) and sucrose (Suc) solutions (lower panel). **B** Tubular and surface membrane capacitance (*C*_t_, *C*_s_) during repetitive measurements compared with capacitance estimated in Tyrode solution (*C*_Tyr_) in a representative experiment. The cell was alternatively exposed to isotonic sucrose and Tyrode solution. Two subsequent evaluations were performed at the end of each steady-state application (50 consecutive current traces were averaged and fitted). Note that the total capacitance (*C*_m_ = *C*_s_ + *C*_t_) measured in the sucrose solution was reduced compared to *C*_Tyr_

If the sucrose solution was washed and reapplied, it was possible to repeatedly estimate these parameters in the same cells, as shown in a representative experiment (Fig. 8). Three repeated applications of the sucrose solution resulted in similar values of *C*_m_, *C*_s_, and *C*_t_. As apparent, *C*_m_ was constantly below *C*_Tyr_, the capacitance measured in the Tyrode solution in the same cell (by ~ 18% on average; see “Discussion” for more details).

## Discussion

Evaluations of tubular membrane capacitance in cardiomyocytes have so far been based on a comparison of the population of detubulated cells with the population of intact cells. The aim of this work was to propose an alternative method that would ensure that the cells remain intact and allow repeated measurements on the same cell. The main idea was based on the assumption that a substantial reduction in the electrical conductivity of the extracellular solution and the associated increase in lumen resistance of the tubular system will make it possible to quantify surface and tubular membrane capacitances (*C*_s_ and *C*_t_) separately using parameters resulting from the double exponential approximation of the capacitive current. To ensure the low conductivity of the extracellular solution, we used an isotonic sucrose solution with the addition of CaCl_2_ at a low concentration (5 µM). Membrane current responses to small voltage-clamped rectangular pulses were analysed to determine the electrical elements of the lumped-parameter model (Fig. 1B).

Membrane capacitances (*C*_s_ and *C*_t_) are considered indicators of membrane areas. Their separate determination is important because the membrane of the tubular system is functionally significantly different from the surface membrane (reviewed by Brette and Orchard [4]). The presence of two capacitances in combination with resistors implies bi-exponential current responses to the imposed steps of membrane voltage. However, in the case of cardiomyocytes in physiological solution, the resistance of the tubular lumen *R*_t_ is very low. This corresponds to the small magnitude and very short time constant of one of the two capacitive current components, which then becomes indistinguishable. In contrast, both components could be distinguished in skeletal muscle fibers, which have a smaller diameter and therefore higher luminal resistance of the tubules [18].

The proposed method is associated with a significant increase in the resistance of the tubular system lumen and the electrical membrane resistance due to the action of the sucrose solution with minimal ionic strength. A question arises as to the nature of the ionic current that remains after replacing the Tyrode solution with sucrose solution. To get a basic idea, we recorded the steady-state current–voltage relations using slow ramp pulses in isotonic sucrose and Tyrode solution for comparison (Fig. 7). The reversal (zero current) voltage was shifted from around − 75 mV in Tyrode to approximately − 140 mV in sucrose solution. The ionic current in the sucrose solution is probably carried by the predominant outward current *I*_K1_ as supported by the effect of addition of Ba^2+^ on the current–voltage relationship (Fig. 7 in [17]). It can be assumed that the inward chloride current *I*_Cl_ controlled by a high positive equilibrium voltage also participates.

The lumped-element model used to describe the membrane system is simplistic. However, simplifications cannot be avoided even in the more complex distributed models. The arrangement of the network of interconnected tubules imaged by microscopic methods in cardiac cells [9, 15, 21] differs from parallel arranged transverse tubules described by cable equations. Moreover, the use of the lumped model is supported by experiments indicating that in rat cardiomyocytes, the tubular length constant λ = (*r*_mt_/*r*_t_)^0.5^ is one order of magnitude larger than the cellular radius [14]. The symbols *r*_mt_ and *r*_t_ denote tubular membrane resistance [Ω m] and resistance of the lumen [Ω m^−1^] per unit of tubular length, respectively. This suggests that the drop of membrane voltage along the transverse tubules can be regarded as negligible so that the tubules are virtually uniformly polarized.

Another simplification is the replacement of voltage-dependent membrane resistances by constants, which corresponds to a linear approximation of the current–voltage relation in the vicinity of the holding voltage (constant slope conductance). Nevertheless, this limitation is minimized by selecting a sufficiently small voltage step for capacitance measurement.

The cell membrane capacitance has been reported to be reduced in skeletal muscle fibres exposed to solutions of low ionic strength [20]. Our results showed an average decrease of the total membrane capacitance in isotonic sucrose solution expressed by the sum *C*_m_ = *C*_s_ + *C*_t_ compared to the capacitance *C*_Tyr_ measured in the Tyrode solution by ~ 18%. Yet, if the decrease in *C*_s_ and *C*_t_ were the same, the coefficient of the fraction of tubular capacitance *f*_t_ (as an indicator of membrane areas ratio) would remain unchanged. Moreover, *C*_m_ and *C*_Tyr_ values are available from repeated measurements on a given cell. Thus, the *C*_s_ and *C*_t_ values can be easily corrected for decreases caused by sucrose solution. The sucrose-membrane interaction has been studied in detail on artificial bilayer membranes in an attempt to explain the effect of disaccharides on membrane stability. Kotowski and Tien [10] observed changes in lecithin membrane properties after exposure to 500 mM sucrose solution. The membrane capacitance was reduced due to a slight increase in membrane thickness caused by sucrose adsorption, which was visible in microscopic observations. Our observation of a reduced capacitance in sucrose solution can be explained within the framework of the so-called water replacement hypothesis which is based on experimental studies and molecular dynamics simulations supporting the concept of a direct sucrose-phospholipid interaction by forming hydrogen bonds to the lipid headgroups [16].

We proposed two different approaches to the approximate determination of tubular membrane capacitance *C*_t_. The advantage of the introduction of the coefficient *γ* defined as the proportionality constant between the ratio of membrane conductances *G*_mt_/*G*_ms_ and membrane capacitances *C*_t_/*C*_s_ is determination of numerical values of all elements of the electrical equivalent circuit of the measured cardiomyocytes (Fig. 1B). These values helped, for example, to determine the accuracy of the method in the publication [17]. The comparison of the results evaluated by both approaches from the same set of measured rat ventricular cardiomyocytes led to virtually the same results. The mean *C*_t_ values differ by 3.5% and the estimate of *C*_t_ determination error was ± 4% in both cases.

The main advantage of the proposed approach is the reversibility of the state of the cells after exposure to a low conductivity solution. Measurements in sucrose and physiological (Tyrode) solution can be alternated several times, as shown in Fig. 8. In comparison with the irreversible detubulation techniques, the proposed approach allows repetitive measurements in the same cell and application of the paired tests. The method could also be useful for separate monitoring of short-term changes in *C*_t_ and *C*_s_ caused by e.g. osmotic shocks [11, 19].

## Methods

### Experimental data

Enzymatic isolation of cardiomyocytes from the right ventricles of adult male Wistar rats and standard experimental procedure using voltage-clamp method have been described previously [1, 17]. Sucrose solution (0.32 M) was prepared by adding sucrose (purity ≥ 99.5%) and CaCl_2_ (5 μM) to deionized water (specific conductivity 1.4 µS/cm). The resulting specific conductivity of the isotonic sucrose solution was 3.7 µS/cm (WTW conductivity meter InoLab Cond 730). The recorded data were evaluated as described in the Results using the following software: Clampfit (v.10.2, Molecular Devices), MATLAB (v.R2017a), and Origin (v.2015).

The accuracy of tubular capacitance determination depends on how thoroughly the tubular system is washed with sucrose solution. The jet pipes for rapid exchange of solutions must be reliably directed at the cell under examination. The magnitude of the change in access resistance can be used as a criterion. A part of the tubular system may be less accessible to sucrose solution if the cell lies at the bottom of the chamber. It is best to lift the cell, which may be however risky. An incomplete exchange of solution will affect the ratio of magnitudes and time constants of both components of the analyzed part of the capacitive current. The unacceptably low resistance of the tubular system lumens will also affect the ratio *R*_1_/*R*_2_ of the resistances calculated according to Eqs. (18) and (19). To decide whether a given measurement is acceptable and can be included in the overall evaluation, we set the following criteria:

28 $$\begin{aligned}{R}_{2}&>20\mathrm{ M\Omega },{R}_{1}/{R}_{2}>0.08,{R}_{\mathrm{a}\_\mathrm{suc}}-{R}_{\mathrm{a}\_\mathrm{Tyr}}\\ &\quad>3\mathrm{ M\Omega }, {J}_{1}/{J}_{2}>0.16, {\tau }_{1}/{\tau }_{2}<10,\end{aligned}$$

where *R*_a_suc_ and *R*_a_Tyr_ are access resistances in sucrose and Tyrode solution, *τ*_1_ refers to the longer of the two time constants.

In all experiments, the capacitive current was approximated by a bi-exponential function using the Clampfit software (Molecular Devices). The resulting values of the parameters *J*_1_, *J*_2_, *J*_∞,2_, *J*_*∞,*1_, *τ*_1_, and *τ*_2_ were then transferred to the software S2_evaluation.mlx provided with all derived computational relationships and necessary procedures for quantifying the parameters of the electrical equivalent scheme. The results of measurements that satisfy the criteria (22) were included in the S2_evaluation.mlx executable file available on request from the corresponding author. The conversion to pdf format is available in  supplementary material.

## Supplementary Information

Below is the link to the electronic supplementary material.

Supplementary file1 (PDF 204 KB)

Supplementary file2 (PDF 98.6 KB)

## Data Availability

The datasets and software written in Matlab language are available from the corresponding author on request.

## Abbreviations

*C*_m_, *C*_t_, *C*_s_: Total, tubular, and surface membrane capacitance (in sucrose solution)
*C*_Tyr_: Total membrane capacitance (in Tyrode solution)
*d*: Membrane thickness
*ε*: Membrane permittivity
*γ*: Coefficient of proportionality between the ratio of membrane capacitances and membrane conductivities
*J*: Membrane current
*J*_1_, *J*_1_: Magnitudes of exponential components of the capacitive current
*J*_∞,1_, *J*_∞,2_: Steady-state currents at the membrane voltages *U*_1_ and *U*_2_
*k*: *C*_T_ /*C*_s_ ratios
*R*_a_: Access resistance
*R*_ms_, *R*_mt_: Membrane resistances
*R*_t_: Tubular system lumen resistance
*S*_s_, *S*_t_: Surface and tubular membrane area
*τ*_1_, *τ*_2_: Time constants of exponential components of the capacitive current
*U*_1_*, U*_2_: Imposed levels of membrane voltage
*U*_s_, *U*_t_: Surface and tubular membrane voltage
