# Supplementary material for: A new approach to the determination of tubular membrane capacitance: passive membrane electrical properties under reduced electrical conductivity of the extracellular solution
Source: Pflugers Arch. 2022 Oct 14;474(12):1263–74. doi: 10.1007/s00424-022-02756-x (PMC9663357; doi:10.1007/s00424-022-02756-x)
Supplement: Supplementary file 1 — Supplementary file1 (PDF 204 KB) [file 424_2022_2756_MOESM1_ESM.pdf]

Supporting information S1_verification

Pflugers Archive - European Journal of Pysiology

A NEW APPROACH TO DETERMINATION OF TUBULAR MEMBRANE CAPACITANCE

Passive membrane electrical properties under reduced electrical conductivity of the extracellular solution

J. Šimurda, M. Šimurdová, O. Švecová, M. Bébarová

Department of Physiology, Phaculty of Medicine, Masaryk University, Brno, Czech Republic

simurda@med.muni.cz

Model verification of the theoretical on of the theoretical background with optional values of the elements of the electrical equivalent circuit (for details see section of Results “Model verification of the theory" in the main text). The 'computational experiments' were designed to mimic the real experiments in isolated cells.

The user has the following options:

- The binary variable F1 determines how the γ-coefficient (gamma) is set. If F1=0, γ is determined by the selected values of the equivalent circuit parameters to meet the condition of Eq (15). If F1=1, γ is to be set (in the interval between 0.4 and 1.2).
- The values of the parameters of electrical equivalent circuit are preset according to Table 2 in the main text but can be changed by the user (in a limited range).

After running the file, graphs of the solution corresponding to figures 2 - 4 (of the main text) will be displayed. The tables at the bottom right show the calculated values of all the elements of electrical equivalent circuit in comparison with the preset values.

```
close all
% clear all
global Ums Umt U1 U2 Too T To tau
global Tt Ts kt kst ket kUt kUs
```

Option: if F1=0, gamma is set to meet condition of Eq. 15; if F=1, gamma can be set arbitrarily in the interval between 0.4 and 1.2

```
% Fl=0;
Fl=1; gamma=1.2;
```

Selected values of the elements of electrical equivalent circuit:

```
% basic choice (nF; MO; mV)
Cs=0.074;     Ra=12.5;
Ct=0.046;     Rt=15;
Ums=-160;     Rms=150;
Umt=-160;     Rmt=241;


% altered values
% Cs=;
%  Ct=0.025;         % smaller Ct must be combined with high Rt
% Ra=38;
% Rt=;
% Rms=;
% Rmt=360;
% Ums=;
% Umt=;
```

Calculation of values of the derived parameters

```
Tt=Rt*Ct; Ts=Ra*Cs; kt=1+Rt/Rmt; kst=1+Ra/Rms+Ra/Rt; ket=Ra/Rt;
kUt=Umt*Rt/Rmt; kUs=Ums*Ra/Rms; ft=Ct/(Cs+Ct);
```

Gamma coefficient

```
if Fl==0
ga=(Cs/Ct)*(Rms/Rmt);
else
    ga=gamma;
end
```

Time constant related to the rising and falling edge of the imposed rectangular pulse

```
tau=0.05;
```

The fitting starts in the instant delayed by *D*t (ms) after the onset of the depolarization step.

```
Dt=0.4;
```

Stimulation protocol

```
T=20; To=30; Too=5;
% T-duration of the voltage impulse, To-time period, % Too-delay

U1=-80; U2=-75;
% holding and impulse voltage
```

SOLUTION OF DIFFERENTIAL EQUATIONS

Initial conditions

```
Us0=(kt*kUs+ket*kUt+kt*U1)/(kst*kt-ket);
Ut0=(kUs+kst*kUt+U1)/(kst*kt-ket);
u0=U1;
```

Computation parameters

```
Tstart=0; Tend=30; InitialStep=0.0000001; MaxStep=0.005; TOL=1e-4;
```

Solution

```
options=odeset('InitialStep',InitialStep,'MaxStep',MaxStep);
[t,y]=ode15s(@model,[Tstart Tend],[Us0; Ut0; u0],options);

 Us=y(:,1);
 Ut=y(:,2);
 u=y(:,3);

 J=(u-Us)/Ra;

 plot(t, u)
 title('Imposed voltage')
 xlabel('Time (ms)')
 ylabel('Membrane voltage (mV)')
 axis([0 30 -81 -74])
 grid on
```

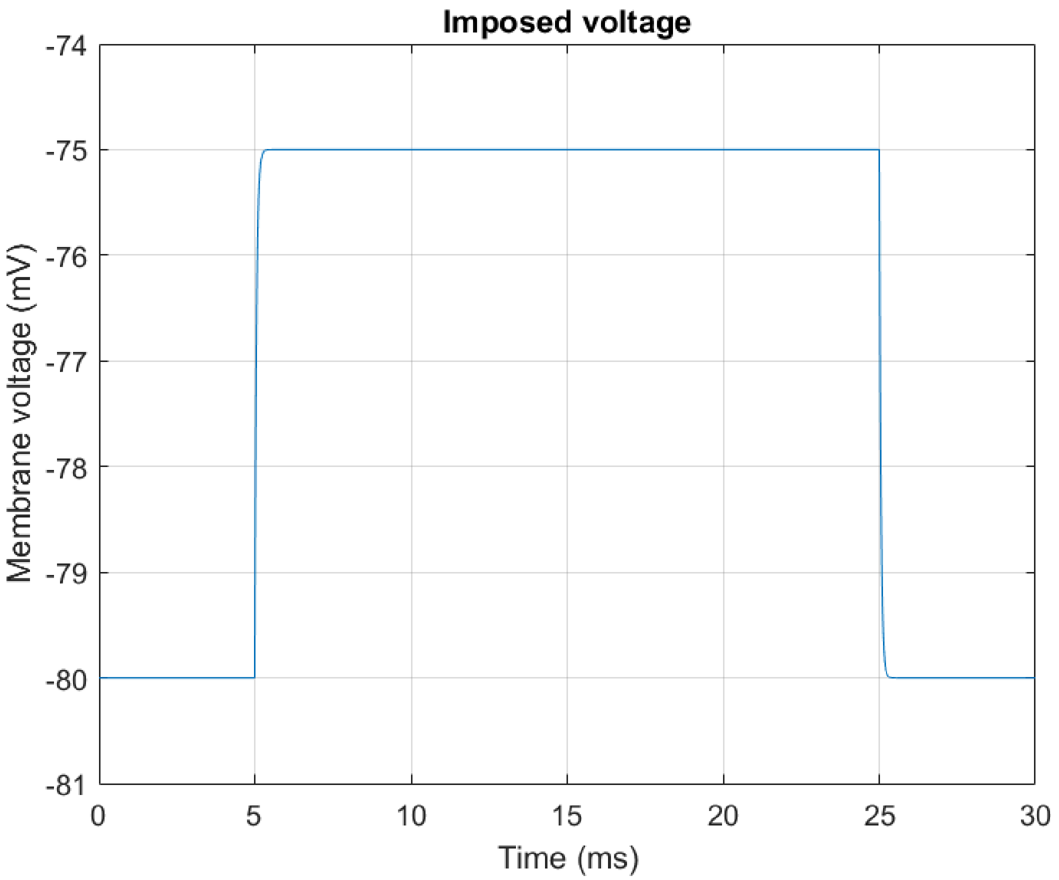


```
 plot(t, Us, t, Ut)
 title('Membrane voltage')
 xlabel('Time (ms)')
 ylabel('Membrane voltage (mV)')
 legend({'surface','tubular'})
%  axis([0 30 -94 -76])
 grid on
```

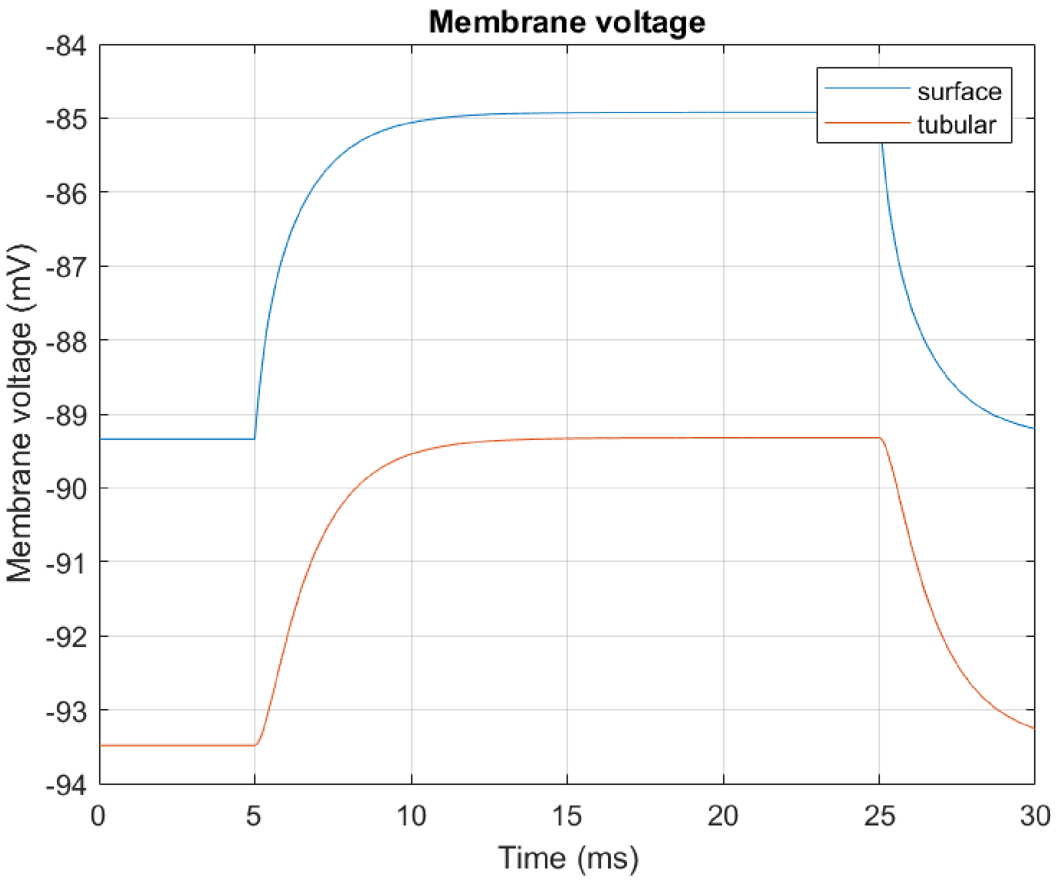


```
plot(t,J,'-')
axis([0 30 0.4 1.15])
title('Capacitive current')
xlabel('Time (ms)')
ylabel('Current (uA)')
grid on
```

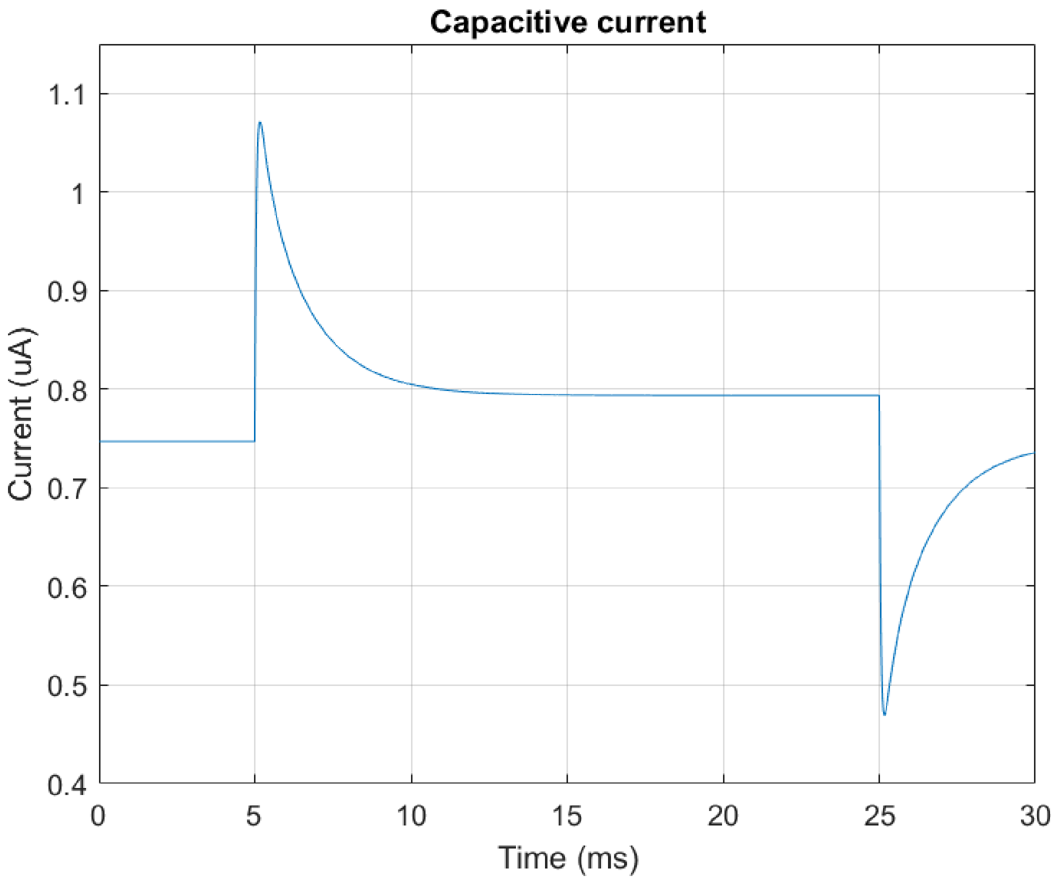

Separation of part of capacitive current for fitting by a bi-exponential function

```
fi=find((abs(t-Too-Dt))<1e-2);
fe=find((abs(t-24.9))<1e-2);
gi=fi(length(fi));
ge=fe(length(fe));
g=gi:ge;
```

Separated part of the capacitive current

```
tt=t(g);
II=J(g);
```

Fitting procedure using Matlab fitting tool

```
 f=fit(tt,II,'J1*exp(-(x-5)/T1)+J2*exp(-(x-5)/T2)+J02', 'StartPoint',...
     [0.251 1.4 0.08 0.34 1.1],...
     'Lower',[0 0 0 0 -Inf]);
```

Parameters resulting from the fitting procedure

```
J1=f.J1;
J2=f.J2;
T1=f.T1;
```

```
T2=f.T2 ;
J02=f.J02;
J01=J(1);
```

Separated part of the capacitive current overlaid by its bi-exponential fit

```
fit_J=J1*exp(-(tt-5)/T1)+J2*exp(-(tt-5)/T2)+J02;

plot(tt, II, tt, fit_J)
% axis([5 25 0.7 1.3])
title('Capacitive current overlaid by its bi-exponential fit')
xlabel('Time (ms)')
ylabel('Current (uA)')
grid on
```

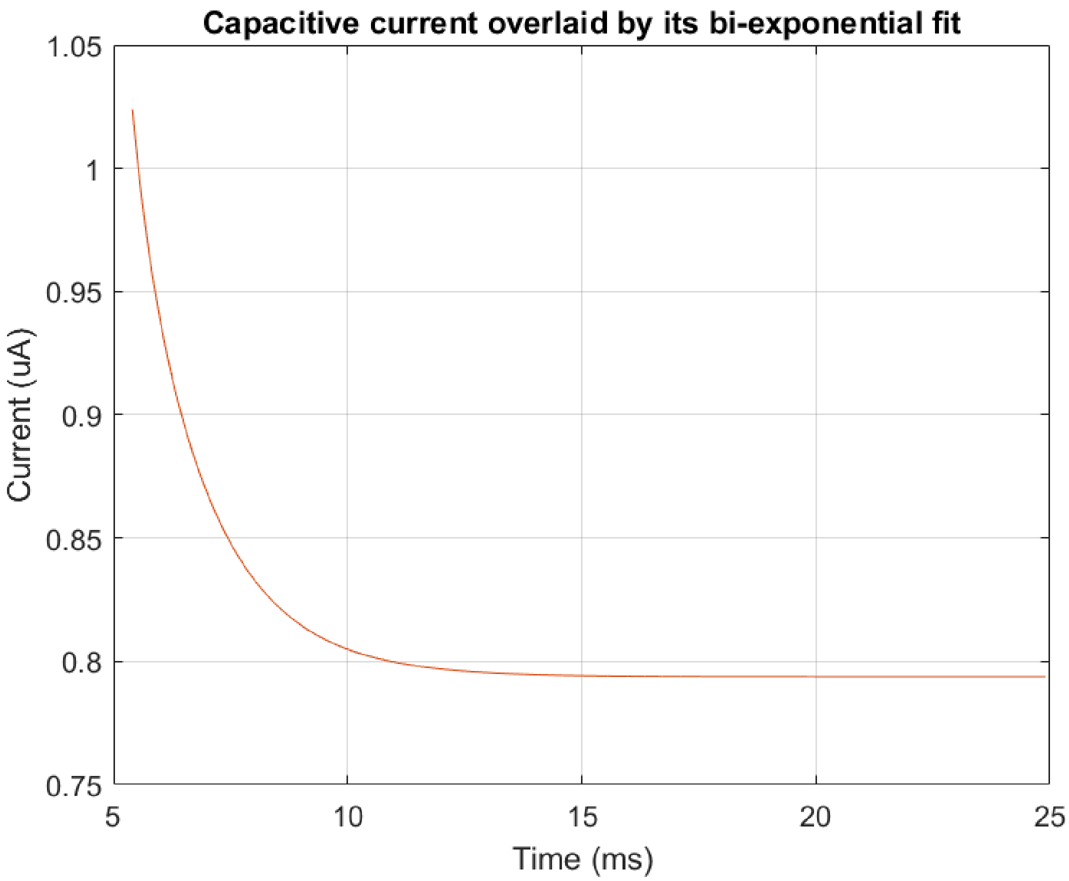

Calculation of some parameters listed in Table 1

The symbol f indicates that the parameter is derived from a bi-exponential fit of capacitive current

```
Raf=(U2-U1)/(J1+J2+J02-J01);
Tsf=(J1+J2+J02-J01)*T1*T2/(T1*J2+T2*J1);
Csf=Tsf/Raf;
a=(J1+J2)/(J1+J2+J02-J01);
b=(T1^2*J2+T2^2*J1)*Tsf/((T1*J2+T2*J1)*T1*T2);
R1=Ra/(b-1);
```

```
R2=Ra*a/(1-a);
Re12=Ra*(T1*J2+T2*J1)/((J1+J2)*Tsf);
```

Calculation of the parameter *k*=*C*t/*C*s:

*G*ms = 1/*R*ms expressed from Eq.17 as a function of the variable *k* (denoted *kk*) is denoted as *G*ms1

```
kk=0.001:0.001:1.8;
Rms1=(R1+R2+ga*kk*(R2-R1)+((R1+R2+ga*kk*(R2-R1)).^2-4*R1*R2).^0.5)/2;
Gms1=1./Rms1;
```

*G*ms = 1/*R*ms expressed from Eq.18 as a function of the variable *k* (denoted *kk*) is denoted as *G*ms2

```
Rms2=((ga*kk-1)*R1*Re12)./(kk*R1-Re12);
Gms2=1./Rms2;
```

Graphic solution of the system of Eqs 17 and 18

```
plot(kk,Gms1,kk,Gms2)
axis([0 0.9 -0.1 0.1])
title('Graphic solution')
xlabel('kk')
ylabel('Gms (uS)')
legend({'Gms1','Gms2'})
grid on
```

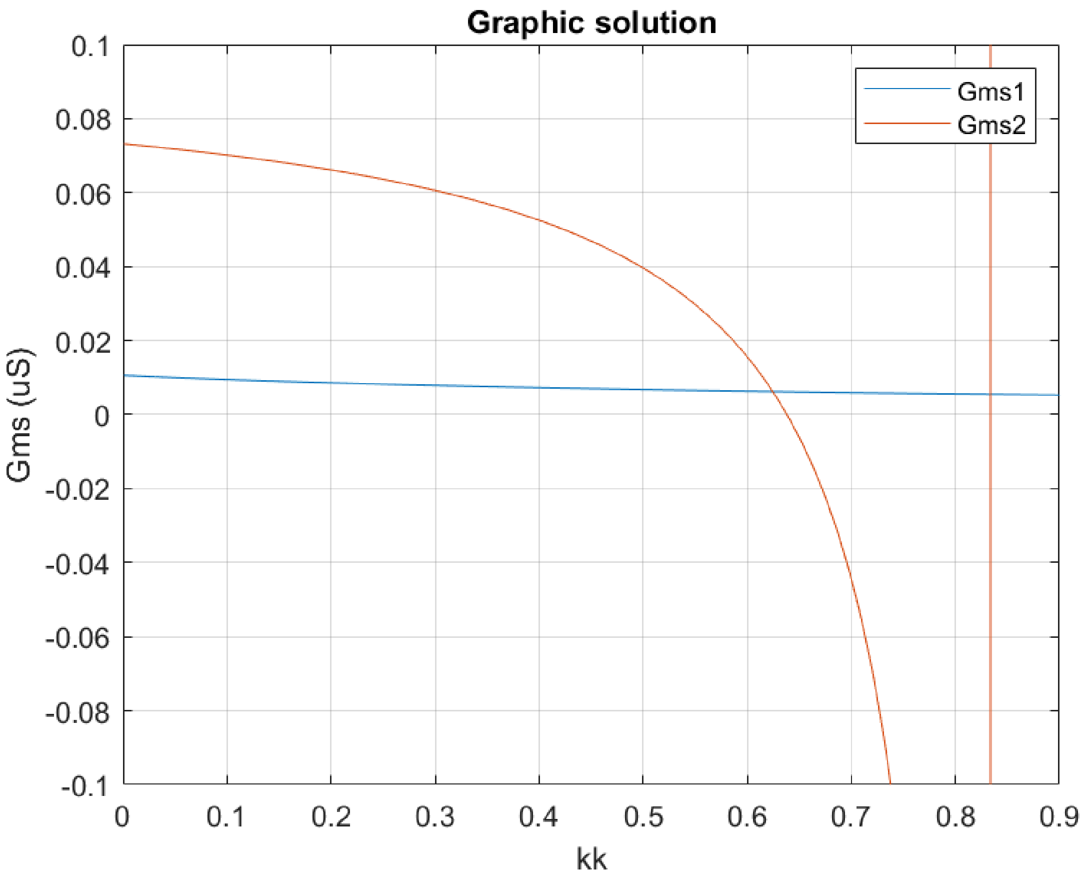

```
% hold on
```

Solution of the system of Eqs.17 and 18 to calculate *k*

```
q=find(abs((Gms2-Gms1))-min((abs(Gms2-Gms1)))==0);
if q>1
Gmsf=Gms2(q(1)); Rmsf=1/Gmsf;
k=kk(q);
```

Calculation of the remaining parameters included in Table 1

```
Ctf=k*Csf;
ftf=Ctf./(Csf+Ctf);
Cmf=Csf+Ctf;
Rtf=R1*Rmsf/(Rmsf-R1);
Rmtf= Rmsf/(ga*k);
 else
     k=NaN; Csf=NaN; Raf=NaN; Rtf=NaN; Rmsf=NaN; Ctf=NaN; ftf=NaN; Rmtf=NaN;
    str=('out_of_range')
 end

 if q==length(kk)
 k=NaN; Csf=NaN; Raf=NaN; Rtf=NaN; Rmsf=NaN; Ctf=NaN; ftf=NaN; Rmtf=NaN;
    str=('out_of_range')
 end
```

Comparison between selected basic parameters and parameters determined according to the proposed method

```
Cs_f=1000*[Cs;Csf]; Ct_f=1000*[Ct;Ctf]; ft_f=[ft;ftf]; Ra_f=[Ra;Raf]; Rt_f=[Rt;Rtf];...
    Rms_f=[Rms;Rmsf]; Rmt_f=[Rmt;Rmtf];
k
```

```
k = 0.6250
```

```
ga
```

```
ga = 1.2000
```

```
Tab1=table(Cs_f, Ct_f, ft_f)
```

Tab1 = 2×3 table

	Cs_f	Ct_f	ft_f
1	74.0000	46.0000	0.3833
2	74.2097	46.3811	0.3846

```
Tab2=table(Ra_f, Rt_f)
```

Tab2 = 2×2 table

	Ra_f	Rt_f
1	12.5000	15.0000
2	12.5339	14.8871

```
Tab3=table(Rms_f, Rmt_f)
```

Tab3 = 2×2 table

	Rms_f	Rmt_f
1	150.0000	241.0000
2	165.6848	220.9131

```
%Ur=U2-J02*Raf/(1-a)%; Ur=U1-J01*Raf/(1-a)
```

Model - solution of differential equations

```
function dydt=model(t,y)
Us=y(1);
Ut=y(2);
u=y(3);

global  U1 U2 Too T To tau
global Tt Ts kt kst ket kUt kUs

if t<Too
   U=U1;
elseif mod(t-Too,To)<=T
     U=U2;
else
     U=U1;
end

dUs=(1/Ts)*(-kst*Us+ket*Ut+kUs+U);
dUt=(1/Tt)*(Us-kt*Ut+kUt);
du=(U-u)/tau;

dydt=[dUs; dUt; du];
end
```
