# Supplementary material for: A new approach to the determination of tubular membrane capacitance: passive membrane electrical properties under reduced electrical conductivity of the extracellular solution
Source: Pflugers Arch. 2022 Oct 14;474(12):1263–74. doi: 10.1007/s00424-022-02756-x (PMC9663357; doi:10.1007/s00424-022-02756-x)
Supplement: Supplementary file 2 — Supplementary file2 (PDF 98.6 KB) [file 424_2022_2756_MOESM2_ESM.pdf]

Supporting information S2_evaluation

Pflugers Archive - European Journal of Pysiology

A NEW APPROACH TO THE DETERMINATION OF TUBULAR MEMBRANE CAPACITANCE

Passive membrane electrical properties under reduced electrical conductivity of the extracellular solution

J. Šimurda, M. Šimurdová, O. Švecová, M. Bébarová

Department of Physiology, Phaculty of Medicine, Masaryk University, Brno, Czech Republic

simurda@med.muni.cz

The experiments on rat ventricular cardiomyocytes and their evaluation were performed using Axon Instruments equipment (Axopatch 200B) and associated software (Clampex and Clampfit). Membrane currents were recorded in sucrose soluton in response to the imposed depolarizing steps of membrane voltage.

Analysis of experimental results: The results of the capacitive current approximation by the sum of two exponential functions obtained by the least squares method in the Clampfit software are available in the first part of this software. In the sample experiments, the capacitive currents recorded in response to 30 ms steps of the membrane voltage from *U*1= -80 mV to *U*2=-70 mV were fitted starting at the instant delayed after the beginning of the voltage step by the time *D*t (given in ms for each measured cell). The parametrs resulting from the fitting process are summarised in the vector MM.

MM vectors in the included experimental results contain numeric values of the bi-exponential capacitive current fit in the following order:

- Magnitude of the first exponential component *A*1 (nA) (*J*1 = *A*1* exp(*D*t/*T*1))
- Time constant of the first exponential component *T*1 (ms); $\tau 1$ in the manuscript
- Magnitude of the second exponential component *A*2 (nA); (*J*2 = *A2* * exp(*Dt*/*T2*))
- Time constant of the second exponential component *T*2 (ms); $\tau 2$ in the manuscript
- Steady state value of current at holding voltage *J*O1 (nA); $J_{\infty,1}$ in the manuscript
- Steady state value of current at the level of imposed step *J*O2 (nA). $J_{\infty,2}$ in the manuscript

In the next part of this software, the data from a selected samlpe experiment are substituted into formulas for calculating the parameters of the electrical equivalent circuit as derived in the article.

The value of the parameter *ga* (corresponding to γ in the manuscript) is optional.

To run the file, it is necessary to "uncomment" the data of the selected cell. Graphical solution of the the system of two equations (17) and (18) appears on the right. Intersection of both curves gives the values of the essential quantities *k* and *Gms*. A table of calculated values of all the elements of electrical equivalent circuit of the selected cell appears at the bottom right.

```
% clc
% clear all

% Parameters from cell number 13205003 - komorové:
MM=[0.471649  1.55114 0.0975374  0.337172 0.4533  0.54379];
Dt=[0.2];

% % Parameters from cell number 13205005 - komorové:
% MM=[0.30438  1.94682 0.0563664  0.499677  0.4291  0.567612];
% Dt=[0.2];

% % Parameters from cell number 132050010 - komorové:
% MM=[0.260789 1.88889 0.0663303  0.41934  0.784  0.978775];
% Dt=[0.2];

% % Parameters from cell number 132050011 - komorové:
% MM=[0.249585 1.91284 0.0633152  0.435593  0.8845  1.06334];
% Dt=[0.2];

% % Parameters from cell number 132050018 - komorové:
% MM=[0.154706 3.37461 0.199835  1.07159  0.9799  1.09261];
% Dt=[0.2];

% % Parameters from cell number 132050019 - komorové:
% MM=[0.271131 2.03963 0.0640055  0.416185  0.9009  1.03691];
% Dt=[0.2];

% % Parameters from cell number 132050044 - komorové:
% MM=[0.415116 1.4444 0.708754  0.487548  0.1773  0.237572];
% Dt=[0.2];

% % Parameters from cell number 132050052 - komorové:
% MM=[0.511943 1.72169  0.261467  0.414618  0.0602  0.109167];
% Dt=[0.1];

% %Parameters from cell number 132050054 - komorové:
% MM=[0.533545 1.53526 0.28271  0.390551  0.0402  0.0816154];
% Dt=[0.1];

% % Parameters from cell number 132050055 - komorové:
% MM=[0.495943 1.5336 0.314228  0.386779  0.0871  0.13702];
% Dt=[0.1];

% % Parameters from cell number 132050064 - komorové:
% MM=[0.37155  2.05961 0.104008  0.339378  0.1817  0.227513];
% Dt=[0.1];

% % Parameters from cell number 132050065 - komorové:
% MM=[0.330961  2.35039 0.148077  0.54016  0.1593  0.22128];
% Dt=[0.1];

% % Parameters from cell number 13219002 - komorové:
% MM=[0.159365 4.962  0.179969  1.21121  0.4827  0.532434];
```

```
% Dt=[0.1];

% % Parameters from cell number 13219003 - komorové:
% MM=[0.0899244  3.05211  0.0857916  0.878286  0.3452  0.406953];
% Dt=[0.2];

% % Parameters from cell number 13219010 - komorové:
% MM=[0.102693 2.526494  0.530023  0.603774  0.4342  0.552734];
% Dt=[0.2];

% % Parameters from cell number 13219011 - komorové:
% MM=[0.081057  3.490718  0.088733  1.015256  0.4774  0.544944];
% Dt=[0.2];

% % Parameters from cell number 13219021 - komorové:
% MM=[0.31474  1.99725 0.0376091  0.347416  0.8214  0.988587];
% Dt=[0.2];

% % Parameters from cell number 13219022 - komorové:
% MM=[0.291017 2.10102  0.0406958  0.335005  0.6925  0.856385];
% Dt=[0.2];

% % Parameters from cell number 13219026 - komorové:
% MM=[0.248671 2.15512 0.0255433  0.300621  0.817  1.00674];
% Dt=[0.2];

% % Parameters from cell number 13219033 - komorové:
% MM=[0.250223  2.13004 0.0351753  0.436589  0.272  0.315922];
% Dt=[0.2];

% % Parameters from cell number 13219039 - komorové:
% MM=[0.245475 2.09377  0.0336863  0.375736  0.2043  0.247449];
% Dt=[0.2];

% % Parameters from cell number 13219045 - komorové:
% MM=[0.501547 1.68712 0.524853  0.546366  0.1779  0.201546];
% Dt=[0.2];

% % Parameters from cell number 13219046 - komorové:
% MM=[0.269419 2.3685 0.0419532  0.368823  0.1886  0.254732];
% Dt=[0.2];

% % Parameters from cell number 13219055 - komorové:
% MM=[0.258325 2.22438 0.0325364  0.319549  0.2311  0.294777];
% Dt=[0.2];

% % Parameters from cell number 13219082 - komorové:
% MM=[0.38664  1.7553  0.0553337  0.3097  0.2626  0.32029];
% Dt=[0.2];

% % Parameters from cell number 13219085 - komorové:
% MM=[0.300362 2.00766  0.0531527  0.34862  0.1823  0.245612];
% Dt=[0.2];
```

```
% % Parameters from cell number 13219091 - komorové:
% MM=[0.328189 1.78957  0.0457228  0.288263  0.4011  0.482341];
% Dt=[0.2];

% % Parameters from cell number 13416005 - komorové:
% MM=[0.372380 2.072792  0.162790  0.474466  0.3065  0.342990];
% Dt=[0.3];

% % Parameters from cell number 13416006 - komorové:
% MM=[0.226340 3.318573  0.188345  0.675546  0.1118  0.133269];
% Dt=[0.3];

% % Parameters from cell number 13416019 - komorové:
% MM=[0.349075  1.554379  0.209296  0.574599  0.2024  0.228173];
% Dt=[0.22];

% % Parameters from cell number 13416021 - komorové:
% MM=[0.253079 2.133046  0.198951  0.618157  0.15  0.171172];
% Dt=[0.25];

% % Parameters from cell number 13416026 - komorové:
% MM=[0.223062 1.743620  0.362101  0.681484  0.3075  0.348136];
% Dt=[0.3];

% % Parameters from cell number 13416027 - komorové:
% MM=[0.157327 2.576585  0.257658  0.925235  0.1861  0.216387];
% Dt=[0.3];

% % Parameters from cell number 13416029 - komorové:
% MM=[0.13654  3.10727 0.264577  0.957714  0.1603  0.186126];
% Dt=[0.3];

% % Parameters from cell number 13416034 - komorové:
% MM=[0.273188 1.933865  0.091846  0.603048  0.2686  0.309869];
% Dt=[0.3];

% % Parameters from cell number 13416035 - komorové:
% MM=[0.297321 1.878515  0.100794  0.482217  0.1741  0.203537];
% Dt=[0.25];

% % Artificial cell (from suppl_1_verification.mlx)
% MM=[0.1794  0.3382  0.5357  1.6285 0.6687  0.7522];
% Dt=[0];
```

CALCULATIONS

Coefficient gamma

```
ga=1.2; %ga=1.0;  ga=0.7;
```

Levels of membrane voltage

```
U1=-80;
U2=-70;
```

Results of bi-exponential fit of capacitive current

```
A1=MM(1); T1=MM(2); A2=MM(3); T2=MM(4); J01=MM(5); J02=MM(6);
```

Current magnitudes extrapolated to the instant of voltage step

```
J1=A1.*exp(Dt./T1);
J2=A2.*exp(Dt./T2);
```

Access resistance (Eq.10)

```
Ra=(U2-U1)./(J1+J2+J02-J01);
```

Time constant related to surface membrane (Eq 11)

```
Ts=((J1+J2+J02-J01)*T1*T2)/((T1*J2)+(T2*J1));
```

Surface membrane capacitance (Eq 11)

```
Cs=Ts/Ra;
```

Calculation of the resistances $R1= Rms\ Rt / (Rms+Rt)$ and $R2 = Rms\ (Rms+Rt) / (Rms+Rmt+Rt)$

(Eqs 12-13)

```
b=(T1^2*J2+T2^2*J1)*Ts/((T1.*J2+T2.*J1)*T1*T2);
R1=Ra./(b-1);
a=(J1+J2)/(J1+J2+J02-J01);
R2=Ra*a./(1-a);
```

Calculation of the resistance $R12$ (defined in Eq 18)

```
Re12=Ra*(T1*J2+T2*J1)/(Ts*(J1+J2));
```

Rms and Gms=1/Rms as a function of variable k (denoted kk) expressed from Eq 17

```
kk=0.05:0.001:1.5;
Rms1=(R1+R2+ga*kk*(R2-R1)+((R1+R2+ga*kk*(R2-R1)).^2-4*R1*R2).^0.5)/2;
Gms1=1./Rms1;
```

Rms and Gms=1/Rms as a function of variable k (kk) expressed from Eq 18

```
Rms2=((ga*kk-1)*R1*Re12)./(kk*R1-Re12);
Gms2=1./Rms2;
```

Solution of the set of Eqs (17) and (18)

```
q=find(abs((Gms2-Gms1))-min((abs(Gms2-Gms1)))==0);
if q>1
k=kk(q);
end
Gms=Gms2(q(1)); Rms=1/Gms;
```

Grafic solution of the set of Eqs 17 and 18

```
plot(kk,Gms1,kk,Gms2)
axis([0 0.9 -0.1 0.1])
title('Graphic solution')
xlabel('kk')
ylabel('Gms (uS)')
legend({'Gms1','Gms2'})
grid on
```

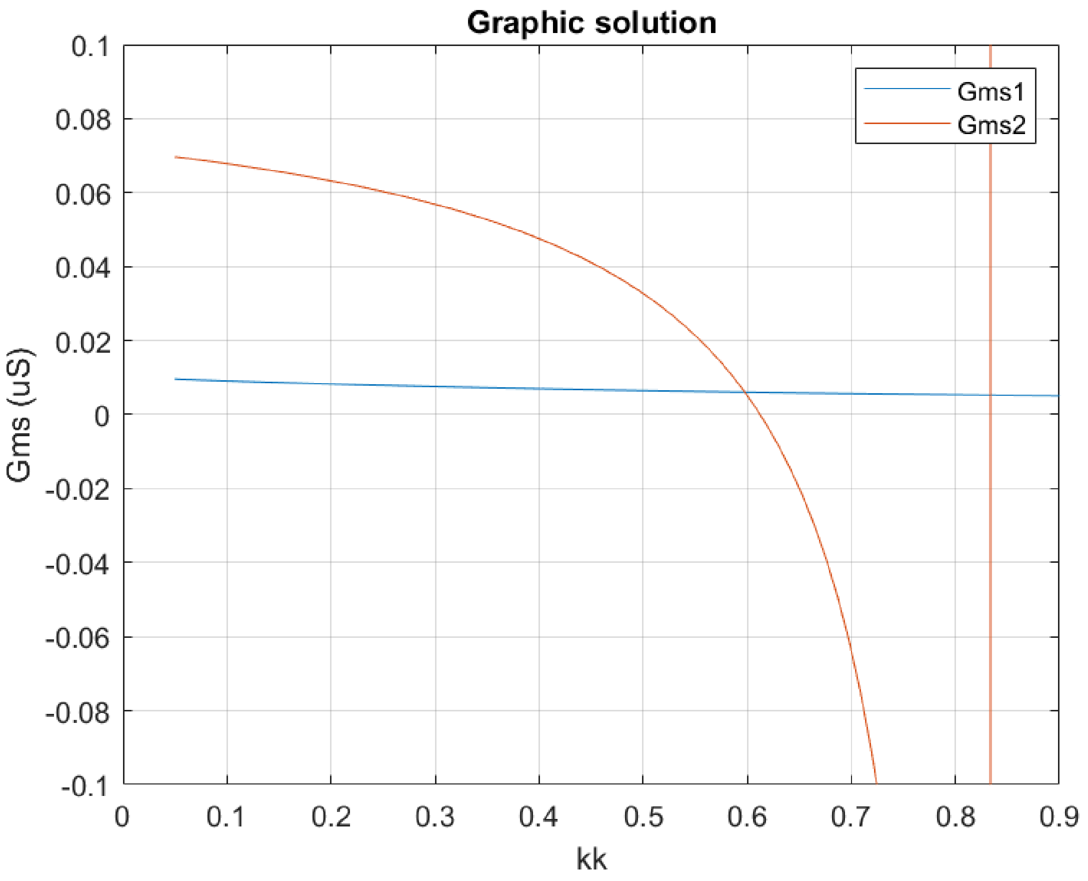


```
% hold on
```

Calculation of *Ct* (Eq 19)

```
Ct=k*Cs;
```

Calculation of the resistances *R*t and *R*mt (Eq 20)

```
Rt=R1*Rms/(Rms-1);   Rmt=Rms/(ga*k);
```

Calculation of the total membrane capacitance and the fraction *ft*=S1/(S1+S2) (Eq 21)

```
Cm=Ct+Cs;    ft=k/(k+1);
```

A rough estimate of the resting voltage *U*r, assuming that its value is the same for the surface and tubular membrane; (to check equality, the value of *U*r is calculated from two relations: Ur1 = Ur2).

```
Ur1=U1-J01.*Ra/(1-a);
Ur2=U2-J02.*Ra/(1-a);
Ur=Ur1;  %Ur=Ur2;
```

Capacitances recalculated from nF to pF

```
Cs=Cs*1000; Ct=Ct*1000; Cm=Cm*1000;
```

Calculated values of the element electrical equivalent circuit (capacitances in pF, resistances in MO, voltage in mV)

```
k
```

```
k = 0.5980
```

```
ga
```

```
ga = 1.2000
```

```
Tab1=table(Cs, Ct, Cm)
```

Tab1 = 1×3 table

	Cs	Ct	Cm
1	74.2686	44.4126	118.6812

```
Tab2=table(ft, Ra, Rt)
```

Tab2 = 1×3 table

	ft	Ra	Rt
1	0.3742	12.4446	14.1040

```
Tab3=table(Rms, Rmt, Ur)
```

Tab3 = 1×3 table

	Rms	Rmt	Ur
1	168.2145	234.4126	-130.0939
